# Pupil engineering for extended depth-of-field imaging in a fluorescence miniscope

**DOI:** 10.1117/1.NPh.10.4.044302

**Published:** 2023-05-08

**Authors:** Joseph Greene, Yujia Xue, Jeffrey Alido, Alex Matlock, Guorong Hu, Kivilcim Kiliç, Ian Davison, Lei Tian

**Affiliations:** a Boston University, Department of Electrical and Computer Engineering, Boston, Massachusetts, United States; b Boston University, Department of Biomedical Engineering, Boston, Massachusetts, United States; c Boston University, Neurophotonics Center, Boston, Massachusetts, United States; d Boston University, Department of Biology, Boston, Massachusetts, United States

**Keywords:** extended depth-of-field, miniscope, computational imaging, fluorescence microscopy

## Abstract

**Significance:**

Fluorescence head-mounted microscopes, i.e., miniscopes, have emerged as powerful tools to analyze *in-vivo* neural populations but exhibit a limited depth-of-field (DoF) due to the use of high numerical aperture (NA) gradient refractive index (GRIN) objective lenses.

**Aim:**

We present extended depth-of-field (EDoF) miniscope, which integrates an optimized thin and lightweight binary diffractive optical element (DOE) onto the GRIN lens of a miniscope to extend the DoF by 2.8× between twin foci in fixed scattering samples.

**Approach:**

We use a genetic algorithm that considers the GRIN lens’ aberration and intensity loss from scattering in a Fourier optics-forward model to optimize a DOE and manufacture the DOE through single-step photolithography. We integrate the DOE into EDoF-Miniscope with a lateral accuracy of 70  μm to produce high-contrast signals without compromising the speed, spatial resolution, size, or weight.

**Results:**

We characterize the performance of EDoF-Miniscope across 5- and 10-μm fluorescent beads embedded in scattering phantoms and demonstrate that EDoF-Miniscope facilitates deeper interrogations of neuronal populations in a 100-μm-thick mouse brain sample and vessels in a whole mouse brain sample.

**Conclusions:**

Built from off-the-shelf components and augmented by a customizable DOE, we expect that this low-cost EDoF-Miniscope may find utility in a wide range of neural recording applications.

## 1 Introduction

Head-mounted miniaturized fluorescence microscopes, or miniscopes, have become an invaluable tool for studying the neural circuits underlying diverse behavior in a capacity that is difficult to replicate in head-fixed animals. By employing off-the-shelf miniature optics and 3D printing, miniscopes uniquely provide a simple, low-cost, and easy-to-adopt neural imaging solution across a wide range of *in-vivo* studies that span timescales between hours and months.^1^^,^^2^ In addition, miniscopes can be surgically implanted by leveraging a gradient refractive index (GRIN) lens to image neural activity at virtually any depth within the brain.^3^ However, many existing miniscopes face an outstanding challenge in terms of the accessible number of neurons within their limited focal region. Due to the use of a high numerical aperture (NA; e.g., 0.5 to 0.55) GRIN objective lens,^2^^–^^4^ only neurons within a narrow (∼15  μm) depth-of-field (DoF) can be imaged with high fidelity. However, neurons are inherently distributed in 3D, which require probing over extended depths to facilitate studies on larger neuronal populations.^4^^,^^5^

In the past few years, several technical advances have enabled the recording of neural activity across extended depths. Miniscopes incorporating an electrically tunable lens (ETL)^6^^–^^9^ enable active focus adjustment; however, the ETL complicates the setup and sacrifices the imaging speed in favor of axial scanning. This leads to an undesirable tradeoff between capturing high-speed neural dynamics and accessing deeper structures. Alternatively, several computational microscopy systems have been developed to record fluorescent signals from an extended depth range. Depending on the final goal, one can classify these techniques into two main categories: “2D-to-3D” and “2D-to-2D.” “2D-to-3D” techniques explicitly reconstruct the 3D fluorescence from a 2D image and enable miniature computational single-shot 3D fluorescence microscopes, such as miniaturized light-field microscope,^10^ Miniscope3D,^11^ Bio-FlatScope,^12^ GEOMscope,^13^ and computational miniature mesoscope,^14^ which encode depth information using a microlens array, customized phase mask or amplitude mask. However, these platforms tend to trade their signal-to-noise ratio (SNR), signal-to-background ratio (SBR), and spatial resolution due to a high-degree of signal multiplexing^15^ as well as require a computationally intensive 2D-to-3D model-based^11^^–^^13^^,^^15^ or deep learning-based^14^^,^^16^ deconvolution algorithm to recover the 3D fluorescence distribution.

Alternatively, “2D-to-2D,” also known as extended DoF (EDoF), techniques “compress” the fluorescent signals from an extended depth into a 2D image by crafting an axially elongated point spread function (PSF) while neglecting the accurate depth information. Previously, several tabletop systems have demonstrated that EDoF imaging techniques can increase the access of neural imaging without the need for costly inverse algorithms.^17^^–^^20^ Typically, the EDoF capability is achieved by pupil engineering, which uses a custom component to modify the optical field as it passes through the pupil plane of a microscope. A large family of pupil functions have been shown to achieve an EDoF.^19^^,^^21^^–^^23^ However, many of these systems rely on active optical devices, such as spatial light modulators, to project the desired function onto the pupil plane of the objective lens. These devices exhibit large form factors and bulky adapters that limit their integration into miniaturized platforms.^24^ Alternatively, diffractive optical elements (DOEs) have been used to directly shape the wavefront at the pupil plane to provide a compact, lightweight alternative for pupil engineering.^25^^,^^26^

**Fig. 1:**
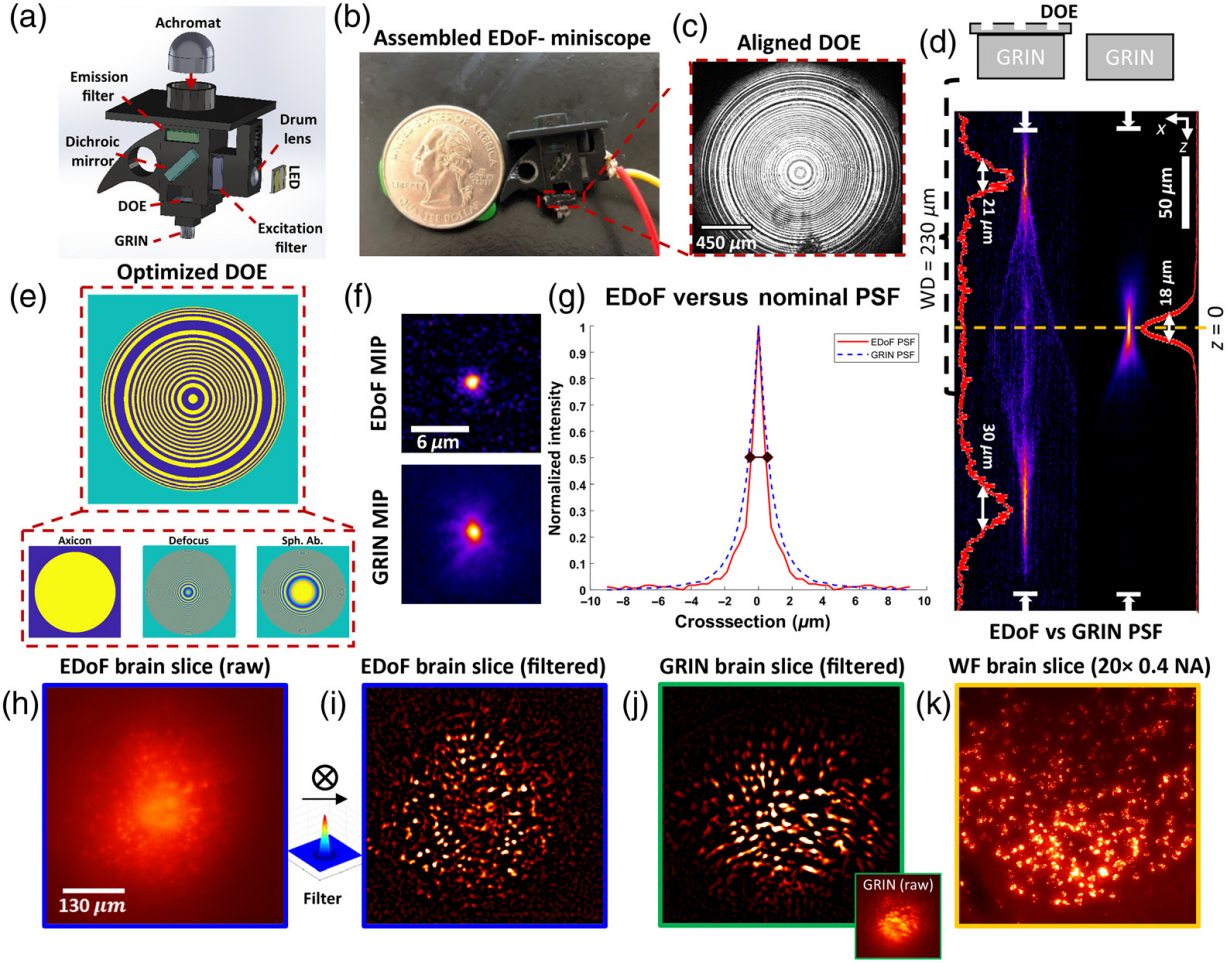
Pupil engineering for EDoF-Miniscope. (a) EDoF-Miniscope combines a lightweight DOE into a miniscope. (b) Picture of EDoF-Miniscope prototype (sensor omitted). (c) Image of the DOE aligned and glued to the back of the 0.55 NA GRIN lens with a collimated laser illumination. (d) XZ cross-section of the 3D PSFs for EDoF-Miniscope (left) and miniscope (right) captured by scanning a 1-μm fluorescent bead on a glass slide through a 346  μm depth range with a 1-μm step size. The GRIN lens working distance (WD; 0.23 mm) allows the utilization of both the +1st (bottom) and −1st (top) diffraction orders created by the DOE to extend the PSF. The full width half maximum (FWHM) of the GRIN PSF is 18  μm and the FWHM of EDoF-Miniscope PSF is 30  μm (+1st order) and 21  μm (−1 order) yielding a 2.8× improvement in the DoF. (e) Breakdown of the genetic algorithm designed phase functions that comprise the DOE. (f) XY MIP of EDoF-Miniscope (top) and miniscope (bottom) PSF over their focal range. (g) Radially averaged cross-sections of EDoF-Miniscope (solid red) and traditional miniscope (dashed blue) PSFs with a FWHM of 0.92 and 1.18  μm, respectively. (h) Raw measurements captured by EDoF-Miniscope of a neuronal population expressing GFP in a 100-μm-thick fixed brain slice imaged through a cover glass (150  μm thick). (i) The same image after postprocessing using our Laplace of Gaussian filter with a standard deviation of 8 pixels. (j) The same region captured by a miniscope before (inset) and after filtering. (k) The MIP image of the same region acquired by a focal stack from a table-top widefield epifluorescence microscope with a 20×, 0.4 NA objective across a 100  μm axial range with a 5-μm step size.

Our goal here is to employ pupil engineering to directly design and integrate a binary phase-only DOE into a miniscope to achieve high-contrast EDoF neural imaging. To this end, we develop EDoF-Miniscope [Figs. 1(a)–1(c)] that extends the recoverable depth range from ∼18 to ∼51  μm [Fig. 1(d)] while maintaining a high spatial resolution when compared to the original miniscope [Figs. 1(f)–1(g)]. We choose to employ a binary DOE to demonstrate the proposed technology so that the pupil element can be easily fabricated by single-step photolithography to facilitate rapid prototyping [Fig. 1(c)]. We optimize our DOE to design twin foci by considering the strong +1 and −1 diffraction order generated by a binary phase DOE to increase our total accessible EDoF without requiring a rapidly oscillatory phase profile and integrate the optimized DOE onto a 230  μm working distance GRIN lens to facilitate interrogating fixed samples with thin cover glasses. By employing a 2D-to-2D encoding strategy, our EDoF-Miniscope only requires a simple filter^27^ for real-time postprocessing for neural signal extraction [Figs. 1(h)–1(k)]. We develop a genetic algorithm to optimize the binary phase mask from a basis of three widely used EDoF phase functions, consisting of axicon, spherical aberration, and defocus^28^ [Fig. 1(e)]. Compared to gradient-based optimization strategies, such as those based on deep learning,^25^^,^^29^^,^^30^ our genetic algorithm allows for more flexible incorporation of nondifferentiable constraints, such as constraining the relative peak intensity over the desired EDoF range and binarizing the phase profile. Our algorithm is built on a linear shift invariant Fourier-optics model and, in addition, incorporates the native spherical aberration of the GRIN lens and the scattering-induced intensity decay. As a result, the optimized binary phase profile achieves an EDoF in addition to the native spherical aberration-induced PSF elongation and is robust to intensity loss due to scattering. Furthermore, the defocus term axially displaces the PSF so that both the +1st and −1st order foci are well separated and within the focal region [Fig. 1(d)]. In this way, our twin foci design remains high contrast when considering other defocused orders and further extends the EDoF range.

We first evaluate the genetic algorithm-designed DOE when integrating with a one-photon (1P) miniscope. Our results show that the fabricated DOE closely matches the simulated profile and can be integrated into an EDoF-Miniscope with a lateral alignment accuracy of 70  μm. We verify that the anticipated amount of lateral and axial misalignment of the DOE on the pupil plane negligibly affects the resulting EDoF performance. The resulting EDoF-Miniscope increases the DoF by ∼2.8× and achieves ∼0.9-μm lateral resolution when tested on 1-μm fluorescent beads. The DOE itself contributes 0.15 g to the total weight and is directly etched on a 3.6  mm×3.6  mm×0.5  mm fused silica substrate, making it a compact solution for pupil engineering in miniscopes.

We experimentally demonstrate EDoF-Miniscope’s imaging capability across a variety of fluorescent samples. We show that EDoF-Miniscope successfully captures an extended fluorescent fibers volume in the presence of a strong out-of-focus background. We characterize EDoF-Miniscope on 5- and 10-μm fluorescent beads embedded in scattering phantoms to demonstrate EDoF imaging in scattering media. We quantify the SBR loss by the DOE across scattering phantoms with different fluorescent bead densities. This proof-of-concept experiment solidifies that EDoF-Miniscope may successfully recover neuron-sized objects in scattering scenes. Finally, we demonstrate the capability of EDoF-Miniscope in several brain samples. First, we image a fixed 100-μm-thick mouse brain sample and compare the EdoF image to a widefield z-stack to show that we recover ∼82% of the total neurons within the volume. Next, we demonstrate EDoF-Miniscope on imaging vasculatures in a fixed mouse brain.

Overall, our contribution is a novel EDoF-Miniscope that achieves EDoF fluorescence imaging by integrating a lightweight DOE into a miniscope. We demonstrate the EDoF fluorescence imaging capability across multiple fluorescent phantoms and brain samples. Built from off-the-shelf components and augmented by a customizable DOE, we expect that our low-cost EDoF-Miniscope may be adopted in many neuroscience labs and find utility in a wide range of neural recording applications.

## 2 Methods

### 2.1 Genetic Algorithm Design and Implementation

We developed the genetic algorithm using the Genetic Algorithm Toolkit in MATLAB 2019b to optimize the DOE over a forward model to minimize a fitness function that encourages the desired EDoF behavior. First, we define a target number of generations, G, for the algorithm to optimize over and select the population size, M, per generation. Next, we define several optical and physical parameters. For the DOE demonstrated in EDoF-Miniscope, we used a scattering length ($l_{s}=100\;\mu m$), refractive index (n=1.33), on-axis (spherical) aberrations for the GRIN lens ($W=W_{040}=29.4$), number of pixels on our pupil plane (N=1000×1000) and properties describing our optical simulation ($\underline{O}$) including the NA of the GRIN lens (0.55), system magnification (9.2×), real space pixel size (3.45  μm), axial step size (1  μm), number of depth planes for the simulated environment (100), and size of our proxy neuron on-axis source (5  μm) to reach a target depth of 80  μm in the first diffraction order. During a single iteration, the genetic algorithm synthesizes a pupil candidate, x, from the current bases, $\overset{¯}{p}$, of EDoF pupil phase functions and generates the binary phase for our DOE using

$$\phi_{\mathrm{DOE}}(u,v)=\pi \times \text{binarize}(\mathrm{axi}(\overset{¯}{p}[1])+\mathrm{defo}(\overset{¯}{p}[2])+\gamma (\overset{¯}{p}[3]),\pi ),$$ (1)

where axi is an axicon phase kernel described by $\pi \overset{¯}{p}[1]\sqrt{u^{2}+v^{2}}$, defo is an angular spectrum defocus phase kernel described by $\frac{2\pi n}{\lambda }\overset{¯}{p}[2]\sqrt{1-(\frac{\lambda u}{n}{)}^{2}+(\frac{\lambda v}{n}{)}^{2}}$, γ is a spherical aberration phase kernel described by $\pi \frac{\lambda }{n}\overset{¯}{p}[3{]}^{4}(u^{2}+v^{2}{)}^{2}$, and (u,v) are spatial frequency coordinates on the pupil plane. The resulting phase is wrapped and binarized to 0 and π to achieve our binary phase. Next, we generate our resulting pupil plane profile by

$$M(u,v)=e^{i\phi_{\mathrm{DOE}}(u,v)},$$ (2)

where M(u,v) is a candidate binary filter for use on the pupil plane of the miniscope. Since our binary phase assumes the values 0 or π, our resulting DOE phase factor will correspondingly assume the values 1 and −1, respectively. Next, the algorithm simulates the slice-wise 3D intensity distribution over the desired depth range by

$$I(x,y;z)=|Ƒ\{Ƒ\{O(x,y;z)\}\times M(u,v)\times A(u,v)\times e^{i(\mathrm{defo}(u,v;z))}\}{|}^{2}e^{-\frac{z}{ls}},$$ (3)

where I(x,y;z) is a 3D intensity distribution, O(x,y;z) is an object function representing a target-sized neuron placed on the optical axis, A(u,v) is the on-axis aberrations from the GRIN lens modeled on the pupil plane as characterized through the use of a Zemax simulation (see Sec. 1.2 in the Supplementary Material), and the last term accounts for the expected intensity loss due to scattering while neglecting the negligible amount of PSF width broadening^31^ within a single scattering mean free path. As a result, each slice in I(x,y;z) represents the optical signal generated by a neuron placed at a distance z in the expected environment. By limiting our simulation to on-axis aberrations, we may utilize a fast and efficient linear shift invariant model, which improves the convergence speed of the algorithm.

After the algorithm determines the cost for each candidate in the current generation, it develops three subpopulations to transfer to the next generation, including the elite children, crossover children, and mutation children. Elite children are replicas of the best performing candidates within a top percentage (in our case 20%) of all fitness values within the current generation, allowing these well-performing traits to transfer unperturbed to the next generation. Crossover children are nonelite children generated by randomly selecting a subset of the population (in our case 4 candidates) and combining the traits of the two best masks within that population. Mutation children are nonelite children generated by the same process as crossover children; however, the traits of each child undergo a random chance to mutate after creation. In our algorithm, each trait undergoes a 10% uniform chance of mutation, where mutation replaces the current value with an argument selected from the allotted bounds. The role of elite children is to keep the best traits unperturbed between generations to enforce that the next generation must contain a fitness value as good as the previous generation. This strategy is known as a “queen bee” genetic algorithm and guarantees global convergence after enough time.^32^ Conversely, crossover children and mutation children are designed to reshuffle the combination of traits in circulation in an effort to discover new optimums without the induced bias of the elite candidates. The crossover fraction (in our case 0.4) determines the fraction of the nonelite children that will be generated through crossover instead of mutation. Once the genetic algorithm selects an optimal mask, we determine the sensitivity of the mask due to lateral displacement and axial displacement on the pupil plane as shown in Sec. 4.1 in the Supplementary Material. For 10 generations with 60 masks per generation, the algorithm typically converges in 80 min. Additional details about the genetic algorithm convergence and completion time are in Sec. 2 in the Supplementary Material.

### 2.2 Design and Characterization of EDoF-Miniscope

EDoF-Miniscope is a standalone miniature fluorescence microscope built from off-the-shelf optical components, a custom DOE and 3D-printed housing as illustrated in Figs. 1(a) and 1(b). EDoF-Miniscope consists of an epifluorescence architecture consisting of an illumination and imaging path to excite and collect fluorescent signals within a circular field of view of ∼600  μm in diameter. The full breakdown for EDoF-Miniscope is in Fig. 1(a). For the illumination path, a surface-mounted light-emitting diodes (LEDs) (Luxeon, Rebel Blue) is aligned to a 3D printed epifluorescent channel in the miniscope. The LED is connected to a driver (LED dynamics Inc., 3021-D-E-350, 350A). The LED illumination first passes through a drum lens (Edmund Optics, 45-549) and is spectrally filtered by the excitation filter (Chroma, bandpass filter, 480/40  nm, 4  mm×4  mm×1.05  mm). The filtered illumination reflects off a dichroic mirror (Chroma, 500 BS, 4  mm×4.8  mm×1  mm) and is focused into the sample through the modified GRIN lens. For the imaging path, we relay the generated fluorescent signal through the modified GRIN lens and spectrally filter the fluorescent signal with an emission filter (Chroma, bandpass filter, 535/50  nm, 4  mm×4  mm×1.05  mm). The filtered signal is magnified 9.2× and focused by an achromatic lens (Edmund Optics, NT45-207, f=15  mm) onto an external monochrome complementary metal-oxide semiconductor (CMOS) camera (FLIR, BFLY-PGE-50A2M-CS). We modeled the imaging path in Zemax to determine the optimal placement of the optics (see Sec. 1.2 in the Supplementary Material).

To integrate the DOE into EDoF-Miniscope, we glue (Noland Products Inc., NOA63) the DOE to the back surface of the GRIN objective lens (Edmund Optics, 64-520) with a 70-μm precision to produce the modified GRIN lens. Next, we place the modified GRIN lens into a 3D printed objective lens holder and adhere it onto our miniscope body by curing dental paste (Pentron, Flow-It ALC, Opaque A1) with a ultraviolet (UV) source (Alonefire, SV003, 10 W 365 nm UV Flashlight). The thickness of the DOE substrate (500  μm) is close to the 230  μm working distance of the GRIN lens allowing us to approximate that the glued DOE is on the back focal plane of the GRIN lens. We confirmed that the lateral and axial placement of our DOE is within our tolerances in Sec. 4.1 in the Supplementary Material. As compared with a miniscope, the assembled EDoF-Miniscope achieves a ∼2.8× improvement in DoF without sacrificing optical resolution.

EDoF-Miniscope is comparable in size and weight to a standard GRIN-based miniscope and incorporates additional features to be demonstrated on a tabletop setup for proof-of-concept (see Sec. 3.2 in the Supplementary Material) with an automated z-sample stage. The top plate is designed to block light leakage from the on-board LED into the external camera and side fin allows EDoF-Miniscope to attach to an O1/2″ Thorlabs (Thorlabs, TR2) post to attach to a linear 1″ XYZ stage (Thorlabs, PT3A). We integrate the modified GRIN lens into an EDoF-Miniscope using a 3D printed objective mount, as shown in Figs. 1(a) and 1(b). With modifications for tabletop use, the resulting EDoF-Miniscope weighs 2.3 g and has a size of 22.39  mm×16.5  mm×15.6  mm. Without these modifications, the platform weighs 1.85 g and has a size of 13.17  mm×8  mm×15.6  mm.

We compare the PSF of EDoF-Miniscope to a miniscope by scanning a 1-μm fluorescent bead (Thermo Fisher Scientific, Fluoro-Max Dyed Green Dry Fluorescent Particles) on a glass slide along z to characterize the DoF of each platform respectively using the automated test setup. As seen in Fig. 1(c), EDoF-Miniscope produces asymmetric imaging foci around the nominal focal plane, as predicted by our Fourier optics-based model. By radially averaging the maximum intensity projection (MIP) of EDoF-Miniscope and miniscope 3D PSFs over their focal range, we may compare their average optical resolution over their imaging range. EDoF-Miniscope produces a comparable resolution to the miniscope, indicating that our optimized PSF does not trade its lateral resolution for the EDoF. The slight differences in the measured resolution is likely due to the manual alignment of the optical components within EDoF-Miniscope and the miniscope, respectively. Noticeably, EDoF-Miniscope’s PSF is characterized by a strong defocus lateral ring, which enables the EDoF behavior and contributes to an increase in background. We can see this increased background by comparing the raw measurement of EDoF-Miniscope on a 10-μm mouse brain slice [see Fig. 1(h)] to the raw measurement from a miniscope [see Fig. 1(j) inlet]. However, Figs. 1(i) and 1(j) show that we recover more signals with EDoF-Miniscope than the miniscope after applying a postprocessing filter to extract cellular bodies with high fidelity (see Methods 2.9, Sec. 6 in the Supplementary Material). We characterize the extraction of the neural bodies between a tabletop widefield system, EDoF-Miniscope, and miniscope in Results Sec. 3.4. This experiment indicates that the DOE is effective at encoding neural signals across an extended depth.

### 2.3 Fabrication of the Binary DOE

**Fig. 2:**
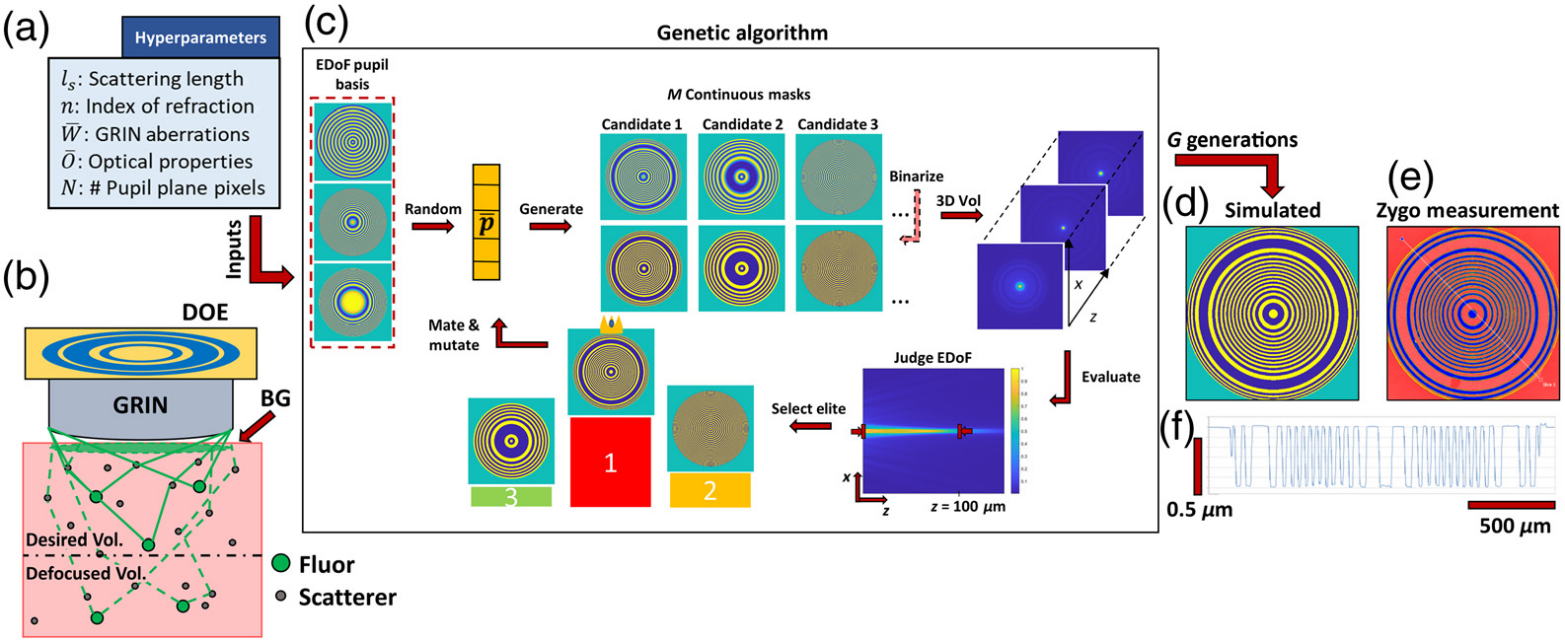
Genetic algorithm for the design of EDoF. (a) User-defined parameters used by the genetic algorithm to optimize the DOE. (b) Overview of the physical model used by the algorithm. We model the scattering effect by an intensity decay that scales with $\frac{z}{l_{s}}$. (c) Overview of the genetic algorithm process. First, the algorithm generates a population of candidate masks from a set of basic phase functions. These candidates are iteratively refined by judging their performance in extending the DoF within the scattering media. (d) Final simulated mask used for EDoF-Miniscope. (e) The final manufactured mask and (f) its cross-section measured by a Zygo New View 9000 interferometer.

We manufactured the binary DOE using single step photolithography in Boston University Class 100 Optoelectronics Processing Facility cleanroom. We first develop a .dxf file containing a 3×5 array of optimized DOEs within a 11.4  mm×19  mm area using AutoCAD and convert the file into a .gdsii using LinkCAD. We upload the .gdsii file onto an optical mask writer (Heidelberg, DWL66) to write the DOE pattern into the photoresist layer of an optical mask blank (Nanofilm, 5X5X.090-SL-LRC-10M-1518-5K). Next, the exposed optical mask is placed in photoresist developer (Microposit, Photoresist Developer MF-319) followed by chromium etchant (Transene, Chromium Etchant Type 1020) for 1 min, which exposes a transparent soda lime glass corresponding to the phase shifted rings in each DOE. The remaining photoresist is removed by submerging the optical mask in a bath of 80°C photoresist remover (Microposit, Photoresist Remover 1165) for 20 min. We next singe a 50.8 mm diameter by 0.500-mm-thick fused silica wafer (University Wafer #971) in a convection oven at 120°C for 15 min to prepare it for pattern transfer. We spin photoresist (Microposit, S1813) onto the wafer at 4000 rpm using a photoresist spinner (Headway Research PWM32-PS-CB15) for 45 s and soft bake the resist at 90°C for 15 min. Next, the wafer and optical mask are aligned using an optical mask aligner (Karl Suss, MA6), and the wafer is exposed the $10\;\mathrm{mW}/{\mathrm{cm}}^{2}$ λ=365  nm UV source onboard for 16.4 s, or until a dose of $∼150\;\mathrm{mJ}/{\mathrm{cm}}^{2}$ was achieved. We added 50  μm of separation between the chrome mask and our wafer to match our desired resolution of 7  μm in accordance with the contact printing equation^33^

$$d=k\sqrt{\lambda h},$$ (4)

where d is the resolution of the photolithography procedure, k is a constant which is $∼\sqrt{2}$ for photolithography, λ is the wavelength of light, and h is the thickness of the photoresist. Next, we develop the mask in a diluted solution of MF-319 and deionized water [2:1] for 75 s to reduce dark erosion and improve the mask quality. After developing, we hardbake the wafer at 120°C for 10 min. Once the photoresist is developed, we etch the final pattern in a reactive ion etcher (Plasma-Therm, Reactive Ion Etcher) using a mixture of ${\mathrm{CHF}}_{3}$ and $O_{2}$ (45-5 sccm) at 40 mTorr pressure for 16.8 min or until the phase shifted rings reach their target depth in accordance with the target phase shift. Reactive ion etching may introduce sideways (isotropic) etching at higher aspect ratios; however, we notice that this effect is negligible except for our outermost ring when we inspect the resulting profile on an optical profilometer (Zygo, New View 9000). The array of DOEs is then aligned to a cutting blade (Disco, DPLU0921) and diced using an automated dicing saw (Disco, DAD 3220). Any remaining photoresist is stripped away in an 80°C bath of 1165 remover. We show a simulated versus manufactured DOE profile in Figs. 2(d)–2(f).

### 2.4 Quantification of Extended Depth Range

To quantify the DoF improvement in experimental results, we use an automated stage to scan a target object across z while being imaged by EDoF-Miniscope or a miniscope. Next, we open the resulting xy image stack in Fiji ImageJ and reslice the array into a stack of xz perspectives. We perform MIP on the xz stack to visualize the 3D optical signal. We extract a line profile detailing the optical signal along the optical axis. We define the recoverable depth range as the FWHM of the optical signal. In practice, this quantity represents the maximum depth range we may observe before the optical signal is reasonably obscured by the high background commonly observed in 1-photon neural imaging.

### 2.5 Scattering Phantom Preparation

We prepared the scattering phantom samples by following the procedure presented in our prior work.^11^ In brief, we embedded 5-μm fluorescent microspheres and a separate sample of 10-μm microspheres (Thermo Fisher Scientific, Fluoro-Max Dyed Green Dry Fluorescent Particles) in a background medium of clear resin (Formlabs, no. RS-F2-GPCL-04; refractive index is ∼1.5403) to act as idealized neurons. We controlled the bulk scattering of the phantom by embedding 1.1  μm nonfluorescent polystyrene microspheres (scatterers) (Thermo Fisher Scientific, 5000 Series Polymer Particle Suspension; refractive index, 1.5979) at a controlled amount. We estimated the effective $l_{s}$ of the phantom using Mie scattering theory

$$l_{s}=-2d\;3\Phi \;Q_{s},$$ (5)

where d is the mean diameter of the scatterers and Φ is the volume fraction of the scatterers. The scattering efficiency, $Q_{s}$, is derived with a Mie scattering calculator. To match a scattering length of neural tissue (100  μm), we add 0.27 mL of a scattering suspension (10% by volume) per mL of clear resin into the phantom. After mixing the suspension with the resin, we leave the resin in a dark location overnight. This period allows the water from the suspension to evaporate away without curing the resin and ensure the background medium remains homogeneous, except for the contribution of the scatterers. The resin is then moved to a glass slide and cured under a high-power UV torch for 30 s.

### 2.6 Whole Fixed Mouse Brain Sample

We prepared the *ex-vivo* whole brain sample by preparing a mixture of gelatin (Sigma Aldritch, G2500) in PBS (ThermoFisher Scientific, 10× pH 7.4, 70011069) diluted 1:10 at 10% weight-per-volume for a maximum amount of 10 ml for one animal. The mixture was placed on a hotplate and heated to 40°C to 45°C to keep the gelatin dissolved. Next a heparinized PBS mixture is prepared by adding 600 iu of heparin (ThermoFisher Scientific, H7482) to 30 ml of PBS and is kept at 40°C to 45°C. Next, 30 mg of fluorescein isothiocyanate (FITC)–albumin (ThermoFisher Scientific, A23015) is added to 1 ml of PBS. Just before perfusion, we filled a beaker with crushed ice and added the FITC–albumin solution to the gelatin solution, shaking gently. Next, we performed a cardiac perfusion with the heparinized saline followed by the FITC–albumin–gelatin. The mouse was put head down into the crushed ice for 15 min. Afterward, the brain was extracted, placed in 4% paraformaldehyde (Electron Microscope Sciences, Diluted 1:8, 15714S) for 6 h, then placed in PBS. To finish the fixation process, the brain in PBS was placed on a horizontal shaker for 3 days.

### 2.7 Zemax Simulation

We conducted a series of Zemax simulations to study the imaging path of EDoF-Miniscope. We used a sequential mode simulation to analyze how the PSF changes as we adjust the placement of the filters and lenses in EDoF-Miniscope’s architecture. We judged the performance of a certain configuration by setting a root mean square spot size merit function at the camera plane when imaging an on-axis and in-focus diffraction-limited spot at our target wavelength. To optimize our design, we performed hammer optimization while constraining the design space so the total lens distances did not exceed the maximum allotted size for EDoF-Miniscope. We analyze how the location of the optics affects the PSF size. We also use our simulation to determine the expected on-axis aberrations generated by EDoF-Miniscope. This process allowed us to optimize the performance of EDoF-Miniscope under our experimental condition and inform our genetic algorithm of the aberrations we need to optimize over. We modeled the geometric effects of the phase mask substrate by adding a 500-μm-thick layer of fused silica at the back surface of the GRIN lens.

### 2.8 Automated Data Collection through Pycro-Manager

We created an automatic data collection pipeline by automating the motion of a Thorlabs single-axis stage (Thorlabs, PT3A) and our scientific CMOS (sCMOS) acquisition using Pycro-Manager.^34^ In brief, Pycro-Manager allows for the seamless control of popular microscopy components and cameras using python routines in an easy to use framework that builds on Micromanager’s core functionality. We captured our diffraction-limited PSF [see Fig. 1(d)] by bringing a dried 1-μm fluorescent bead (Thermo Fisher Scientific, Fluoro-Max Aqueous Green Fluorescent Particles) on a glass slide into focus using our experimental setup. Next, we used our Pycro-Manager stage to scan the sample in z with a 1-μm step size from −150 to 150  μm. We set the exposure time to maximize the in-focus signal without saturation using the camera with 16-bit discretization; however, the framerate never fell below 30 Hz. We repeated the procedure for our 5- and 10-μm bead-scattering sample with a 1- and 5-μm axial step size, respectively. We used Pycro-Manager to automatically apply a filter to each captured frame to save both a raw and filtered stack during an acquisition.

### 2.9 Postprocessing Filter Design and Implementation

Our filter closely follows a filter design proposed for extracting fluorescent speckle signals from a low contrast measurement,^27^ which takes the form

$$f(x,y;\sigma )=-\nabla^{2}\frac{1}{2\pi \sigma^{2}}e^{\left(-\frac{\left(x^{2}+y^{2}\right)}{2\sigma^{2}}\right)}\;.$$ (6)

This kernel, known as the Laplacian of Gaussian (LoG) kernel, reduces noise sensitivity in a measurement while highlighting rapid changes in intensity in a single, easily precalculated kernel. Although commonly used in edge detection,^35^ prior work^27^ showed that adapting the standard deviation of the kernel to the approximate size of the fluorescent object provides targeted background suppression while reducing noise. We confirm the properties of the LoG kernel in Sec. 6.1 in the Supplementary Material. We apply the filter to our simulated data using MATLAB and to our experimentally collected data in Python. In both instances, we determine the optimal σ, as explored in Secs. 6.2 and 6.3 in the Supplementary Material, and preallocate the Fourier transform of the LoG kernel for easy deployment. Afterward, we may rapidly apply the kernel to process the target measurement using minimal computation resources.

## 3 Results

### 3.1 Genetic Algorithm-based Design of DOEs on EDoF-Miniscope

To design a DOE for deployment in 1P neural imaging, we develop a genetic algorithm. Our algorithm employs a linear shift invariant Fourier-optics model to optimize a binary DOE from a basis of three EDoF phase functions. In addition, we consider the native aberrations of the GRIN lens, as well as the exponential intensity decay due to tissue scattering. In this section, we highlight main insights into the algorithm design and refer to implementation details in Methods 2.1, Sec. 1.1–1.3 in the Supplementary Material.

A key feature of EDoF-Miniscope image formation is the compression of axial information from an extended 3D volume to a 2D image. This requires that the optimized PSF must maintain a high contrast over an extended depth range to remain distinguishable from the out-of-focus background present in 1P neural imaging.^1^ However, EDoF PSFs often achieve their “nondiffractive” properties at the expense of strong sidelobes that lowers signal contrast in dense scenes.^36^ We address this issue using a fitness function that judges the DOE based on how effectively it maintains peak intensity over the desired depth range, which reinforces the PSF to maintain a sharp contrast over the desired EDoF. In addition, we design the algorithm to optimize the EDoF PSF on neuron-sized objects (5 to 10  μm) by judging the resulting optical signal after convolving the 3D PSF with a simulated on-axis neuronal source at each depth. This allows the algorithm to further consider the geometric extension from finite-sized objects along with the diffractive effects from the binary DOE. We limit our analysis of the GRIN lens aberrations to third order on-axis seidel aberrations since higher order terms are dominated by high spatial frequencies on the pupil plane and the GRIN lens exhibits strong spherical aberration. At the end of each generation, our genetic algorithm refines the population by producing “children” masks that contain a mix of properties from the best masks in the current generation. By repeating this process over a few generations, the algorithm optimizes the desired EDoF pupil function. We analyze the convergence of the algorithm as a function of population size and number of generations in the Supplementary Material. We experimentally show that the resulting EDoF-Miniscope maintains a comparable SBR to a miniscope and that our designed PSF effectively elongates the PSF across the entire FoV for neural sized objects in Secs. 5.1–5.3 in the Supplementary Material.

The input to the algorithm includes the scattering length ($l_{s}$), refractive index (n), on-axis aberrations for the GRIN lens ($\overset{¯}{W}$), number of pixels on our pupil plane (N), and properties describing our optical simulation ($\overset{¯}{O}$) including the NA of the GRIN lens, system magnification, simulated FoV size, axial step size and number of depth planes for the simulated environment, and size of our proxy neuron on-axis source [see Fig. 2(a)]. Within each “generation,” the algorithm optimizes over a set of basic phase terms, including axicon phase, defocus, and spherical aberration, to design a population of DOEs by solving the minimization problem, as illustrated in Figs. 2(b) and 2(c)

$$\min_{\overset{¯}{p}⋴B}f(I(x,y,z;\overset{¯}{p})),$$ (7)

where $I(x,y,z;\overset{¯}{p})$ is the resulting 3D (x,y,z) intensity profile of the PSF parameterized by the pupil phase basis, $\overset{¯}{p}$, as determined by our forward model described in Sec 1.1 in the Supplementary Material. B is the user-defined optimization bounds for $\overset{¯}{p}$. The fitness function f judges the quality of the EDoF PSF generated by each candidate within the population and takes the form

$$\text{cost}=-\underset{z\in z_{\text{target}}}{\sum }\text{binarize}(\frac{I(x,y;z)}{I_{\max}},0.5)+\alpha \underset{z\notin z_{\text{target}}}{\sum }\text{binarize}(\frac{I(x,y;z)}{I_{\max}},0.5),$$ (8)

where $z_{\text{target}}$ is the desired extension range, which is chosen based on the scattering length that sets the practical imaging depth limit for 1P fluorescence imaging. The operator binarize(g,0.5) binarizes the 3D function g using 0.5 as the threshold, which effectively reinforces the desired EDoF PSF to retain at least 50% the maximum intensity $I_{\max}$ over the desired depth range across all depths. To prevent the algorithm from overextending the EDoF, we penalize any intensity profiles outside of the desired depth range by the $z\notin z_{\text{target}}$ term. α, which we heuristically set equal to 4, is a tunable parameter that determines the softness of the bound.

After optimization for a scattering length of 100  μm and refractive index of 1.33, our binary mask is parameterized by a strong spherical aberration term, a strong defocus term, and a negligible axicon contribution. By the binary nature of our mask, the +1st and −1st diffraction orders receive conjugate versions of the learned phase. It is important to note that each order will be subjected to the same underlying aberrations, which may be modeled as a continuous pupil phase on top of our binary mask. In the +1st order, our mask adds an additional 26.65 waves of spherical aberration, which exaggerates the native spherical aberration of the GRIN lens to greatly extend the focal range but behind the nominal focal plane. To compensate the defocus displacement of 129  μm to bring the EDoF in front of the nominal focal plane. In the −1st order, the learned −26.65 waves of spherical aberration partially cancels the native aberration of the GRIN lens and displaces the focus by −129  μm to keep the order behind the nominal focal plane. As a result, the +1st order is more extended than the −1st order. In addition, the opposing defocus terms allow each order to produce nonoverlapping axial profiles, so that our total DoF can be considered as the sum of each respective FWHM. Altogether, our optimized binary mask is able to produce larger EDoF through our twin foci design, as shown in Fig. 1(d). We further demonstrate our genetic algorithm’s flexibility to design DOEs to achieve an EDoF under different scattering conditions and refractive indices in Sec. 7 in the Supplementary Material. Our genetic algorithm learns different weights of axicon, defocus, and spherical aberration under each respective condition showing that the optimal binary mask varies as a function of the chosen physical parameters.

We select an optimized mask [Figs. 2(d)–2(f)] and demonstrate its utility in imaging neural signals in brain tissues after being integrated into EDoF-Miniscope. We manufactured the mask using the single-step photolithography (see Methods 2.3) and aligned the DOE to the GRIN lens using the process described in Sec. 3.1 in the Supplementary Material. Practical considerations for integrating the mask, such as the effect of lateral and axial misalignment on the resulting EDoF, are further studied in Sec. 4.1 in the Supplementary Material.

### 3.2 Experimental Demonstration on a Thick Fluorescent Fibers Sample

**Fig. 3:**
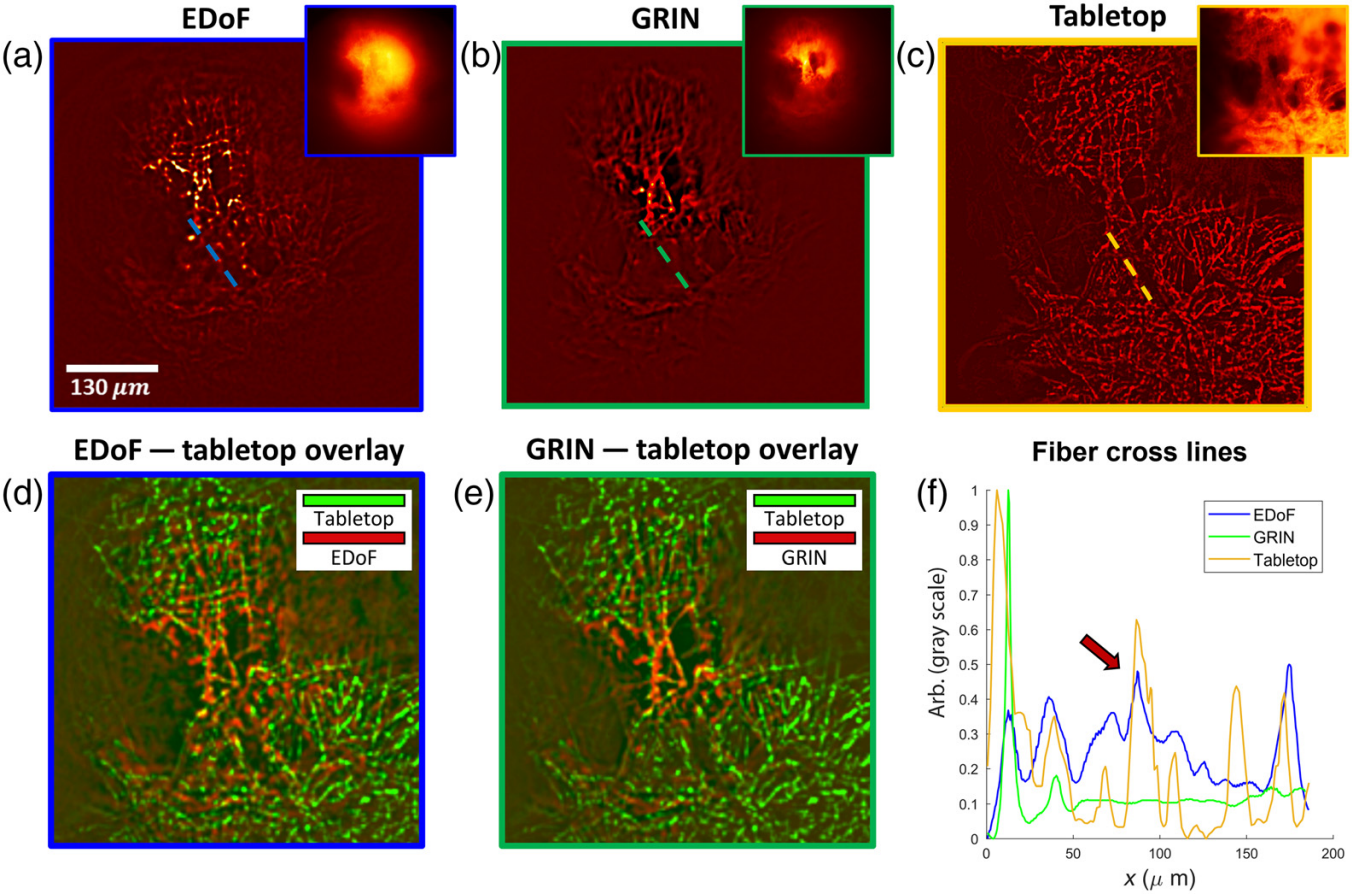
Extended depth of field in a fluorescent fiber sample. (a) A fluorescent fiber sample imaged by EDoF-Miniscope before (inlet) and after filtering. (b) The same sample imaged by the miniscope and (c) MIP from widefield images collected over 125  μm with a 5-μm step size. (d) Overlay of the tabletop widefield MIP (green) and a single frame from the EDoF-Miniscope (red) after postprocessing. (e) Overlay of the tabletop widefield MIP (green) and a single frame from a miniscope (red) after postprocessing. (f) Cutlines across the same fiber bundle over the three previous images. The arrow indicates a region where EDoF-Miniscope matches the synthesized widefield image but the miniscope is defocused.

We experimentally verify the ability of EDoF-Miniscope on a thick complex fluorescence object by imaging fluorescent-stained fibers spread on a glass slide. The sample spans the full FoV (∼600  μm×600  μm) and an extended depth (400  μm) to test the imaging performance of EDoF-Miniscope when subjected to a strong out-of-focus background. EDoF-Miniscope raw measurement [see Fig. 3(a), inlet] exhibits a lower contrast when compared with the miniscope raw measurement [see Fig. 3(b), inlet]; however, this is due to the extended imaging range as well as an increased background. The SBR for EDoF-Miniscope raw measurement is ∼1.1 compared with 1.26 for the miniscope. As shown in Fig. 3(a), EDoF-Miniscope can clearly recover closely packed fiber structures across the full FoV after applying the postprocessing filter to the raw data. The EDoF is highlighted by comparing the image to a miniscope [see Fig. 3(b)]. Visually, EDoF-Miniscope is able to recover fiber structures over sections of the FoV that are defocused in the miniscope image.

To qualitatively show EDoF-Miniscope’s EDoF capacity, we compare both images to an MIP obtained by projecting a z-stack of images collected by a tabletop widefield setup across 125  μm with a 5-μm step size [see Fig. 3(c)] using an objective lens (Nikon, CFI Plan Fluor, 10×, 0.25 NA). We reinforce that the postprocessed images exhibit consistent features across the full 2D image by presenting overlays between the tabletop widefield MIP as well as the EDoF-Miniscope [Fig. 3(d)] and miniscope images [Fig. 3(e)], respectively. However, we notice that both miniscope images tend to degrade toward the peripheries. Since the postprocessing filter leverages subtle contrasts to extract features, its performance qualitatively degrades due to signal attenuation caused by off-axis aberrations. Notably, EDoF-Miniscope retains optical signals over a larger portion of the FoV than the miniscope, indicating that EDoF is partially resilient to off-axis aberrations. We further explore the off-axis properties of EDoF-Miniscope in Secs. 5.1 and 5.2 in the Supplementary Material. In addition, we plot a cutline across a fiber bundle between each image, as shown in Fig. 3(f). EDoF-Miniscope retains more fibers than the miniscope when compared to the widefield MIP. The differences in the relative intensity scaling of the cutline between each image are due to the different illuminations used during the measurements (manually aligned epi-illumination for the miniscopes versus on-axis for the 10× objective lens) and aberrations.

This experiment highlights the capability of EDoF-Miniscope to extract fluorescent signals across the full FoV in the presence of an out-of-focus background. The complex and thick geometry of the fibers verifies that EDoF-Miniscope may encode and extract arbitrarily shaped fluorescent sources through a simple filter to suppress any increased background. As a result, EDoF-Miniscope can provide a high quality EDoF with good robustness to background signals.

### 3.3 EDoF in a Controlled Scattering Phantom

To further consider EDoF-Miniscope toward neural imaging applications, we examine the performance of EDoF-Miniscope when encoding neuron-sized (∼5 to 10  μm) sources under bulk scattering. To do so, we conduct experiments on fluorescent bead phantoms with scattering properties similar to that of neural tissue. Our phantom has a scattering length of ∼100  μm and an anisotropic factor of ∼0.97. We embed 5-μm fluorescent beads as proxy neurons in the scattering phantom to showcase the robustness of EDoF-Miniscope to scattering media in this proof-of-concept experiment.

**Fig. 4:**
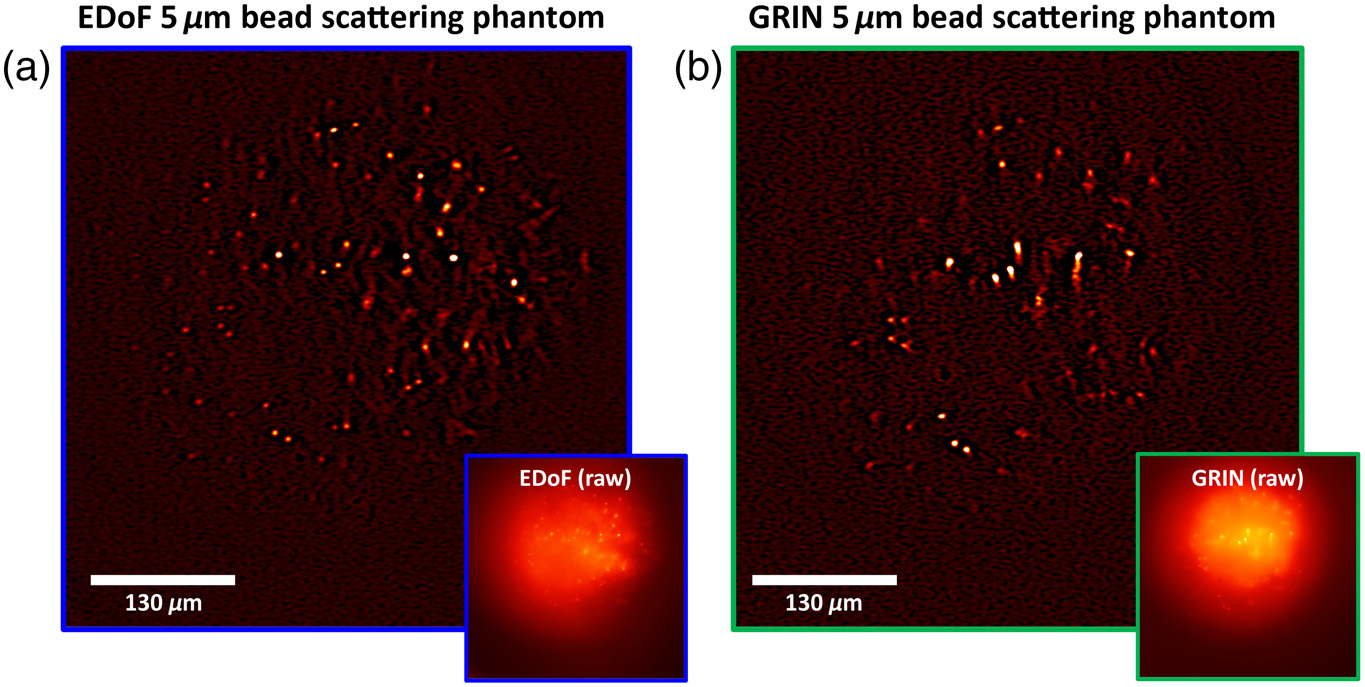
EDoF characterization in scattering phantoms. (a) EDoF-Miniscope image of 5-μm beads embedded in clear resin with 1-μm polystyrene scatterers ($l_{s}=100\;\mu m$) before (inlet) and after a filter. (b) The same region imaged by a miniscope, before (inlet) and after filtering.

We image a spherical cap-shaped phantom (diameter = 2.2 mm, depth = 0.65 mm) embedded with 5-μm fluorescent beads at a density of $∼2120\;\text{particles}/{\mathrm{mm}}^{3}$ (see Methods 2.5). Visually, EDoF-Miniscope raw image [see. Fig. 4(a), inlet] exhibits less contrast in the center of the FoV but retains more particles at the peripheries of the FoV when compared to the miniscope raw image [see. Fig. 4(b), inlet]. After filtering, our [see Figs. 4(a) and 4(b)] EDoF-Miniscope successfully extracts 111 particles versus 66 particles with the miniscope within the FoV. On an average, EDoF-Miniscope image exhibits an SBR of 1.06, whereas the miniscope exhibits an SBR of 1.10. We further explore the differences in SBR as a function of particle density in Sec. 5.3 in the Supplementary Material and conclude that EDoF-Miniscope trades on average a ∼4% decrease in SBR for its EDoF when imaging fluorescent bead samples (∼1000 to $10,000\;\text{particles}/{\mathrm{mm}}^{3}$). We characterize the axial elongation of the 5-μm beads by both EDoF-Miniscope and miniscope to judge the achievable imaging depth when interrogating nondiffraction limited, neuron-sized sources. We accomplished this by axially sweeping a sparse scattering phantom with a custom-automated sample stage (see Sec. 3.2 in the Supplementary Material). EDoF-Miniscope achieves an imaging depth of 104  μm between both foci versus an imaging depth of 37  μm with the miniscope in Sec. 5.1 in the Supplementary Material. We designed the first order to have an elongation of 80  μm for 5-μm-sized objects in scattering tissue; however, here, we observe an elongation of 67  μm. We predict that this discrepancy is due to the GRIN lens having a lower spherical aberration than what is predicted in our Zemax model. We repeat the procedure for 10-μm fluorescent beads and characterize the elongation on several locations across the FoV (see Sec. 5.2 in the Supplementary Material) to show that EDoF-Miniscope also elongates off-axis sources, even if only on-axis aberrations are considered in our optimization.

### 3.4 Imaging Neuronal Structures in Brain Slices

**Fig. 5:**
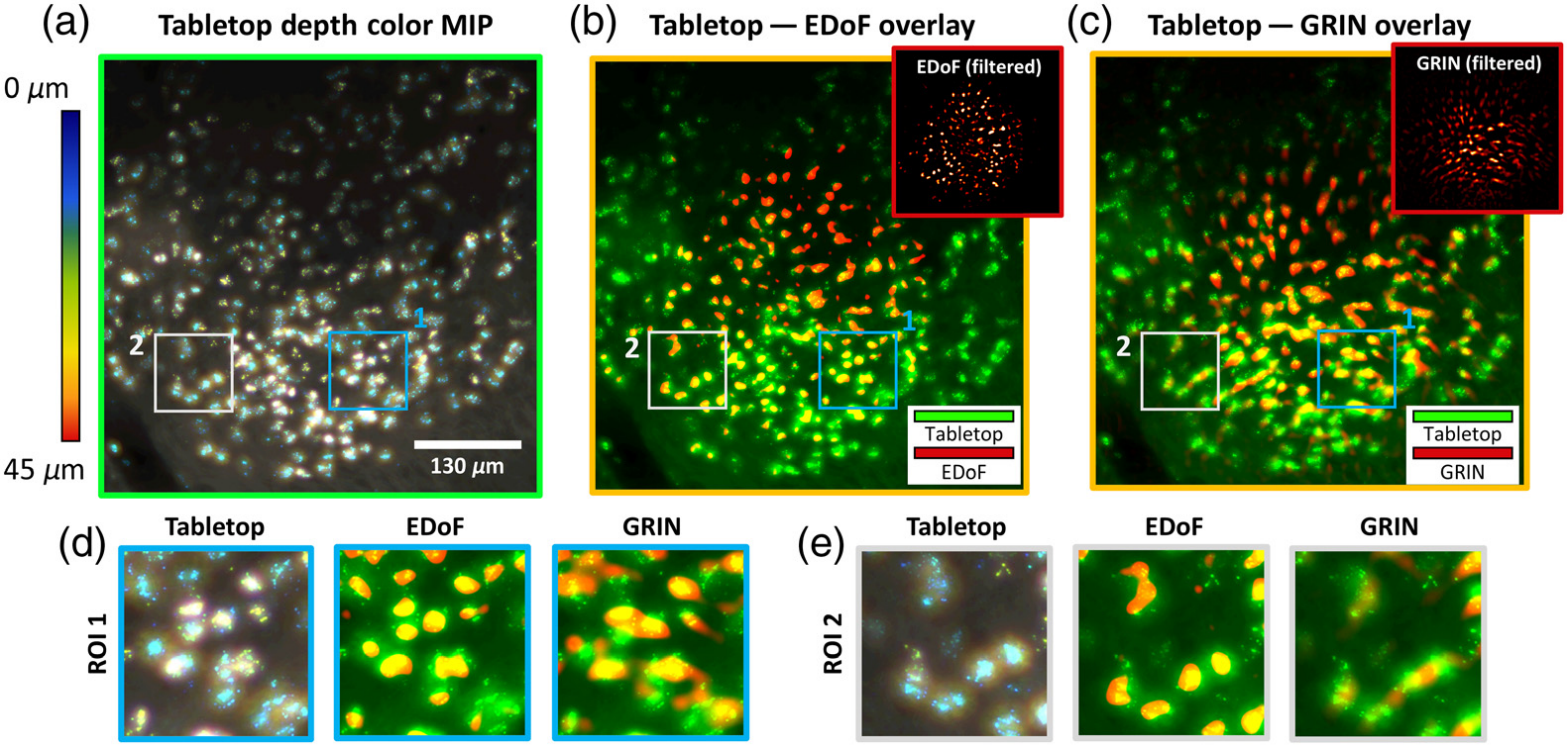
Confirming extraction of neuronal structures. (a) Tabletop MIP of a neuronal population within a thin brain slice captured over a 45  μm depth range with a 5-μm step size. (b) Overlay of the tabletop MIP and the filtered single shot EDoF-Miniscope frame. (c) Overlay of the tabletop MIP and the filtered single shot miniscope frame. (d) Zoom in on the same ROI between the tabletop, EDoF-Miniscope overlay, and miniscope overlay near the center of the FoV. (e) Zoom in of the same ROI between the tabletop, EDoF-Miniscope overlay, and miniscope overlay near the peripheries of the FoV.

To confirm the capacity of EDoF-Miniscope to accurately extract neuronal structures, we image the same neuronal population of a 100-μm-thick fixed brain slice stained with green fluorescent protein (GFP) in an axial sweep with a widefield tabletop imaging system [see MIP in Fig. 5(a)] and in a single-shot with an EDoF-Miniscope with [see Fig. 5(b)] and without postprocessing filtering [see Fig. 5(b), inlet]. The widefield stack was acquired on a sCMOS camera (PCO.Edge 5.5, pixel size=6.5  μm) with a Nikon 20× 0.4 NA objective by performing a z-scan 100  μm depth range with a 10-μm step size. Visually, both EDoF-Miniscope image and widefield MIP capture neuronal structures in the center of the FoV; however, the widefield MIP has better performance near the peripherals due to the less extreme off-axis aberrations in the objective lens. Despite this disparity, EDoF-Miniscope recovers 174 neurons in a single shot and the widefield MIP recovers 213 neurons over its z-scan range.

Next, we overlay the images to perform additional visual inspections. We resample the widefield MIP to the same discretization as the EDoF-Minsicope image and crop the images to the same FoV. We confirm that the structures extracted in the filtered EDoF-Miniscope frame match well with the neuronal structures recorded in the widefield MIP. This result confirms that our framework can properly extract neurons from the background without introducing major artifacts. It is important to note that the widefield MIP and EDoF-Miniscope images are not in perfect agreement across the whole FoV. This variation is due to misalignment in the miniaturized imaging system combined with distortions in the miniaturized optics that are not accounted for in the overlaying process. It is also important to note that the EDoF-Miniscope image exhibits slightly worse performance in the center of the FoV when compared with the miniscope image. By considering the raw images [Figs. 1(h) and 1(j) (inlet)], we notice that the EDoF raw image exhibits a stronger background (as predicted by Sec. 5.3 in the Supplementary Material) especially at the center of the FoV. This lowers the contrast of cells in that area and reduces the fidelity of the postprocessing filter. However, these factors only affect the neuronal recovery mildly, and visual verification is still possible across the FoV. We additionally visually verify that filtering performs superior neuronal extraction than direct deconvolution with morphological background removal for EDoF-Miniscope image in Sec. 6.2 in the Supplementary Material.

### 3.5 EDoF Imaging of Vasculatures in a Fixed Whole Mouse Brain

**Fig. 6:**
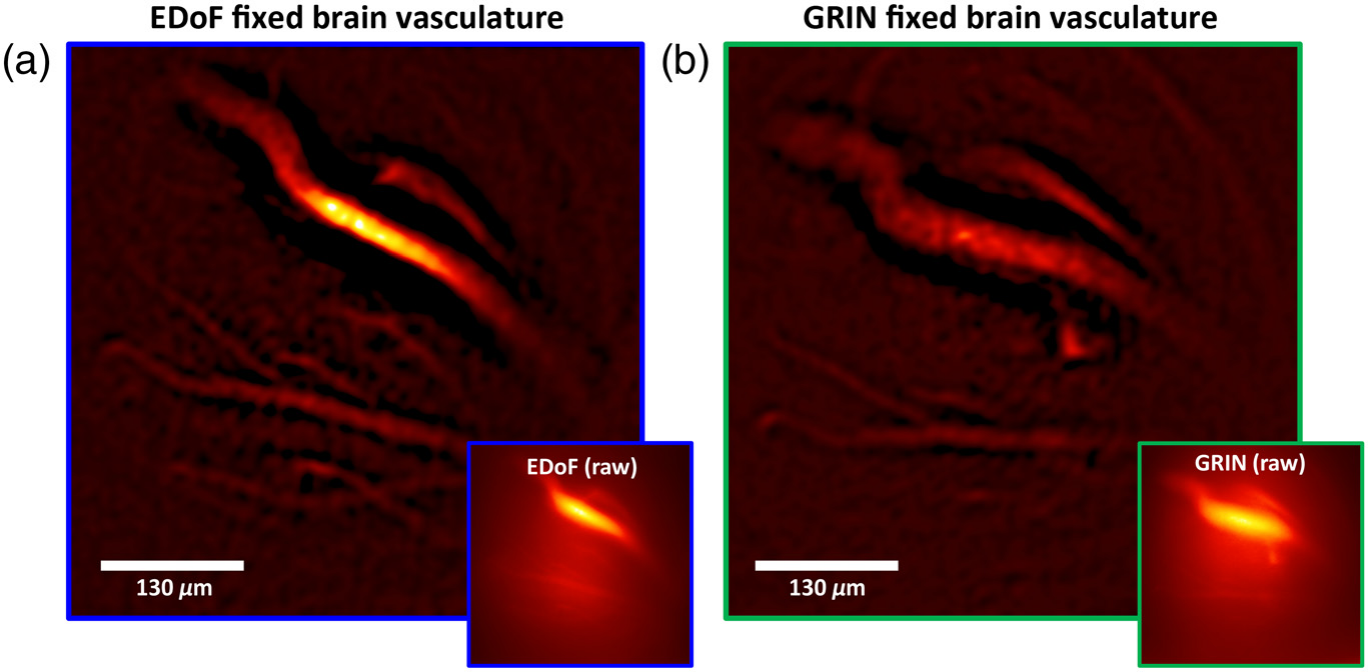
EDoF imaging of vessels in a fixed mouse brain. (a) Fluorescently stained vessels in a whole fixed mouse brain captured by a EDoF-Miniscope. EDoF-Miniscope was placed such that the pronged vessel was centered in the DoF. (b) The same region captured such that the pronged vessel was in-focus on a miniscope.

We imaged fluorescently stained vessels in a fixed whole mouse brain sample. As shown in Figs. 6(a) and 6(b), we imaged the same vasculatures under both EDoF-Miniscope and a miniscope such that the pronged vessel in the center of the FoV was in focus. This central vessel exhibits an SBR of 1.09 for EDoF-Miniscope and 1.11 for the miniscope. Visually, both raw measurements are sparse allowing us to directly inspect the structures. Filtering allows us to analyze the fine features and inspect the relative sizes of the vessels. A further comparison of using our postprocessing filter versus deconvolution with morphological background removal can be found in Sec. 6.3 in the Supplementary Material. The miniscope image retains fewer capillaries and exhibits a broadening of the large vessel when compared with EDoF-Miniscope image. This result highlights that EDoF-Miniscope is able to capture diverse types of fluorescent objects.

## 4 Discussion and Conclusions

In summary, we have presented a novel EDoF-Miniscope, which applies a binary phase DOE to augment a miniscope for EDoF neural imaging. The system uses a genetic algorithm to design an aberration-informed and scattering-robust EDoF for deployment in 1P fluorescent neural imaging to achieve a ∼2.8× improvement in the DoF when compared to a standard miniscope. We experimentally verified EDoF-Miniscope across a variety of complex fluorescent and neuronal samples.

The main contributions of EDoF-Miniscope include its novel design and integration of a binary DOE into a miniscope to achieve the single-shot recording of extended neural signals without sacrificing spatial resolution. The genetic algorithm synthesizes a DOE over the native aberrations of the miniscope by refining a pool of candidates from a basis of three EDoF phase functions to optimize a scattering-robust EDoF PSF. After manufacturing, the DOE itself weighs only 0.15 g and may be integrated into an EDoF-Miniscope with an accuracy of 70  μm. EDoF-Miniscope exhibits a ∼4% decreased SBR compared with a standard miniscope. We use a simple filter for postprocessing to extract extended fluorescent signals across the full FoV.

Our pilot demonstration on the utility of DOEs in miniscope imaging may be a particularly attractive area for future research as it augments the open source miniscope and encourages customizability as well as supports rapid development for use across a diversity of experiments. By promoting an easily manufacturable and customizable DOE and requiring minimal modification to the miniscope architecture, EDoF-Miniscope allows researchers to optically tailor their miniscopes to match their experimental requirements. Broadly, we expect that the synergy between physics-based computational design strategies and customized miniature phase masks will continue to improve the interrogation of neural signals in miniaturized microscopes and endoscopes with additional novel capabilities, such as extended FoV.^37^

In its current prototype, EDoF-Miniscope is designed for performing proof-of-concept experiments in fixed samples in a tabletop setup. In future applications, we intend to replace our 230  μm working distance GRIN lens with a 0 working distance GRIN lens, which is more suitable for *in-vivo* studies.^38^ We will also use a backside illuminated (BSI) CMOS sensor to significantly improve the SNR and dynamic range for *in-vivo* studies. Although cutting-edge sensors may increase the size and weight of the miniscope,^15^ we are encouraged by the recent development of the MiniFAST BSI CMOS-based miniscope^39^ in successfully applying high data rate and high pixel count miniaturized BSI sensors to future generations of EDoF-Miniscope. Our studies indicate that we may further decrease the size and weight of the platform while nominally affecting its imaging properties to further reduce the formfactor (see Sec. 1.2 in the Supplementary Material).

In future iterations of the project, we intend to investigate three promising directions to improve our results. First is using our framework to design binary DOE for zero working distance GRIN lenses. By leveraging a zero working distance lens, we suppress the −1st order of diffraction, thus pressuring our design to maximize the utility of the 1st order of diffraction. We explore some potential optimizations in Sec. 7.1 in the Supplementary Material. In addition, proper characterization of our GRIN lens will play a crucial role in ensuring that our designed EDoFs will be accurately predicted by our simulation. In exploring our Zemax GRIN lens model, we notice some potential sources of model mismatch, which we present in Sec. 7.2 in the Supplementary Material. Finally, we seek to explore how we might encourage our genetic algorithm to learn a single contiguous EDoF by merging the twin foci together. In some preliminary simulations, we demonstrate this capability by more cleverly synergizing our twin foci design. These optimized DOEs may offer the potential to achieve an EDoF beyond one scattering length, which we present in Sec. 7.3 in the Supplementary Material.

An outstanding challenge in expanding EDoF-Miniscope toward *in-vivo* studies is overcoming the high background present in *in-vivo* neural imaging. There are several promising solutions we envision for future generations of EDoF-Miniscope, such as replacing the binary DOE with a miniature refractive element,^11^^,^^37^ incorporating structured illumination techniques,^6^^,^^40^ and advanced computational techniques to facilitate neuronal signal extraction.^41^^–^^44^

## Code, Data, and Materials Availability

The genetic algorithm implementation and postprocessing algorithms are available at: https://github.com/bu-cisl/EDoF-Miniscope.
