# Supplementary material for: Pupil engineering for extended depth-of-field imaging in a fluorescence miniscope
Source: Neurophotonics. 2023 May 8;10(4):044302. doi: 10.1117/1.NPh.10.4.044302 (PMC10197144; doi:10.1117/1.NPh.10.4.044302)
Supplement: Supplementary file 1 [file NPh_010_044302_SD001.pdf]

Contents

1 Optical Modeling & Characterization 2

1.1 Forward Model Implementation 2

1.2 System Characterization in Zemax 3

2 Genetic Algorithm Convergence 5

3 Optical Setups 6

3.1 High Precision DOE Alignment Setup 6

3.2 Automated Test Platform for Fixed Samples 8

4 DOE Perturbation Analysis 8

4.1 Effect of Lateral and Axial DOE Displacement on the DoF 8

4.2 Alignment Precision under Visual Guidance 10

4.3 Phase Shift Error versus Deployed EDoF 10

5 EDoF Characterization on Fluorescent Samples 12

5.1 Axial Elongation Within a 5 µm Bead Scattering Phantom 12

5.2 Axial Elongation Within a 10 µm Bead Scattering Phantom 13

5.3 SBR Analysis Over 5 µm Bead Phantoms of Varying Density 14

6 Analysis of Post-Processing Filter 14

6.1 Investigating Filter Properties 14

6.2 Investigating Filter Performance on Simulated 5 µm Sources 15

6.3 Investigating Filter Performance on Simulated 10 µm Sources 16

6.4 Comparison to Deconvolution - Brain Slice Sample 17

6.5 Comparison to Deconvolution - Vasculature 18

7 Exploration of DOE Optimization Under Different Conditions 18

7.1 DOE Optimization for Single Focus Extension 19

7.2 Exploration of Model Mismatch 20

7.3 DOE Optimization of Twin Foci to Push Possible EDoFs 20

7.4 Learned DOE Parameters 22

1 Optical Modeling and Characterization

1.1 Forward Model Implementation

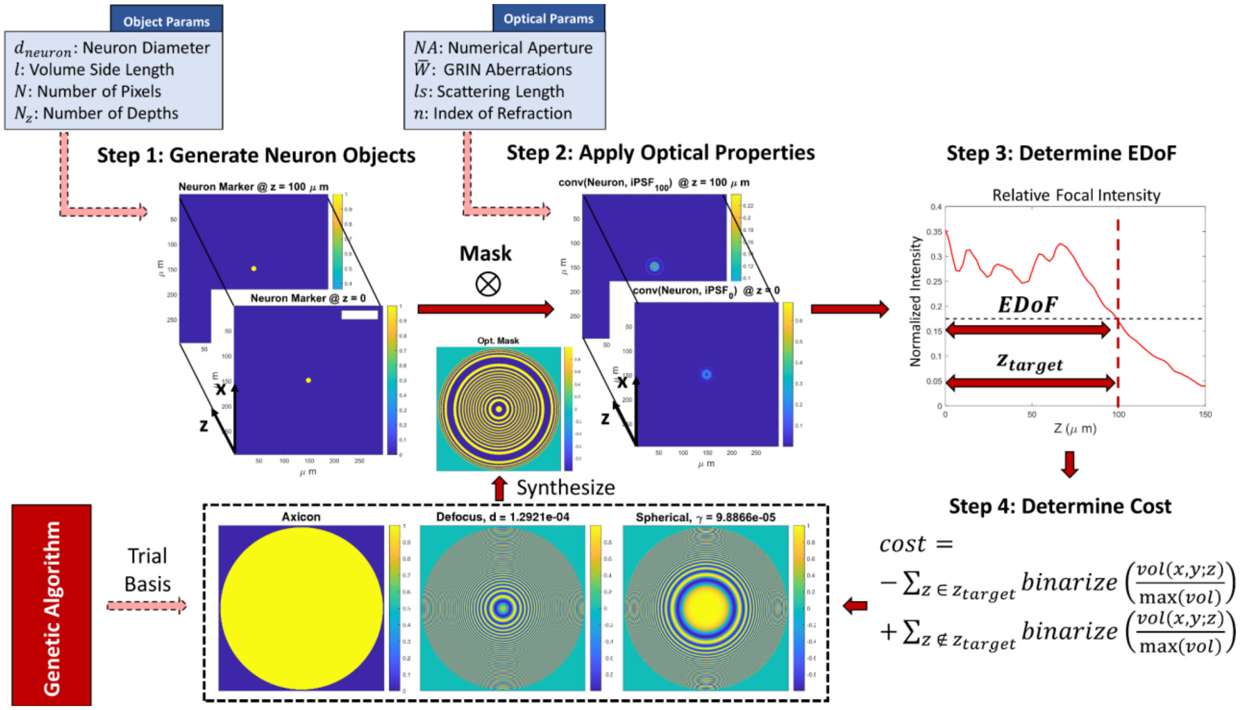

Fig S1.1. Genetic algorithm forward model. The genetic algorithm selects a candidate and generates the 3D optical signal for an on-axis neuron-sized object. The forward model leverages user-defined information to generate an informed image. After generating the 3D signal, our algorithm judges the EDoF on the full-width-half-maximum of the on-axis XZ cross-section. Applying the fitness function to each candidate allows the algorithm to refine the current population and produce children for the next generation.

The imaging model used in this work follows a scalar diffraction approximation to describe the light emitted from an isotropic fluorescent emitter[45]. Our optical platform is a 4f system with a diffractive optical element placed on the pupil plane. We assume that the emitters are suspended in a user-defined media parameterized by an average refractive index, n_m, and a scattering length, l_s. We assume that the media is strongly forward scattering, as is common with biological tissue[31]. Under these assumptions, we may characterize the optical signal resulting from an on-axis neuronal source on our image plane by:

$$I(x,y;z) = |F\{F\{o(x,y;z)\} \times M(u,v) \times D(u,v;z) \times A(u,v)\}|^2 \times e^{-z/l_s}, \quad \text{(S1)}$$

where *(x,y)* is our spatial coordinates, *z* is the current depth relative to the focal plane, and *(u,v)* are the corresponding spatial frequencies. $o(x,y;z)$ is our on-axis object function, which we typically assume is a 5 μm circle as proxy neurons. To characterize the EDoF, we simulate on-axis emitters where the thickness of our fluorescent sources matches our axial resolution. The volume is

described by hyperparameters set by the user before running the simulation (see **Methods 2.1**). $M(u, v)$ is the pupil function modulated by our d binary mask placed on the pupil plane. $D(u, v; z)$ is the angular spectrum defocus kernel and takes the form:

$$D(u, v; z) \ = \ e^{ikz\sqrt{1-(NA/n_m)^2}}, \tag{S2}$$

where k is the wavevector, $NA = \lambda\sqrt{u^2 + v^2}$ is the pupil coordinates relative to the numerical aperture, and n_m is the index of refraction of the propagation media. $A(u, v)$ is the on-axis Seidel aberrations characterized by our Zemax simulation (see **Supp. 1.2**). Since we are only considering third order on-axis aberrations, this term consists solely of spherical aberration. The trailing term is the expected intensity decay due to scattering.

Once we receive an optimized DOE, we may formulate a simulated widefield image of a neuronal scene with a modified version of S1:

$$I_{im}(x, y) \ = \ \sum_z \quad |F\{F\{o_{im}(x, y; z)\} \times P(u, v; z)\}|^2 \times e^{-z/l_s} + b(x, y) + \eta(x, y), \tag{S3}$$

where $o_{im}(x, y; z)$ is now randomly distributed emitters on each slice and $P(u, v; z)$ is the combined effects of the DOE, defocus and aberrations on the pupil. To simulate the strong out of focus background common in 1P neural imaging, $o_{im}(x, y; z)$ contains both 5 μm target emitters and 1 μm background emitters that provide background fluorescence[15]. We additionally add a background term, $b(x, y)$, which is a user defined Gaussian function with a set waist and maximum value to produce the desired SBR in the simulated image. $\eta(x, y)$ is Gaussian noise applied to the resulting image that simulates the effects of sensor noise. When the optical signal exceeds 5 photons per pixel, shot noise can be well approximated as Gaussian noise and all common noise sources, excluding fixed pattern noise and flicker noise, can be combined into a single Gaussian noise source[46]. For the sake of simplicity, we assume that our sensor produces an ideal flat field so we neglect the effects of hot pixels and fixed pattern noise in simulation.

1.2 System Characterization in Zemax

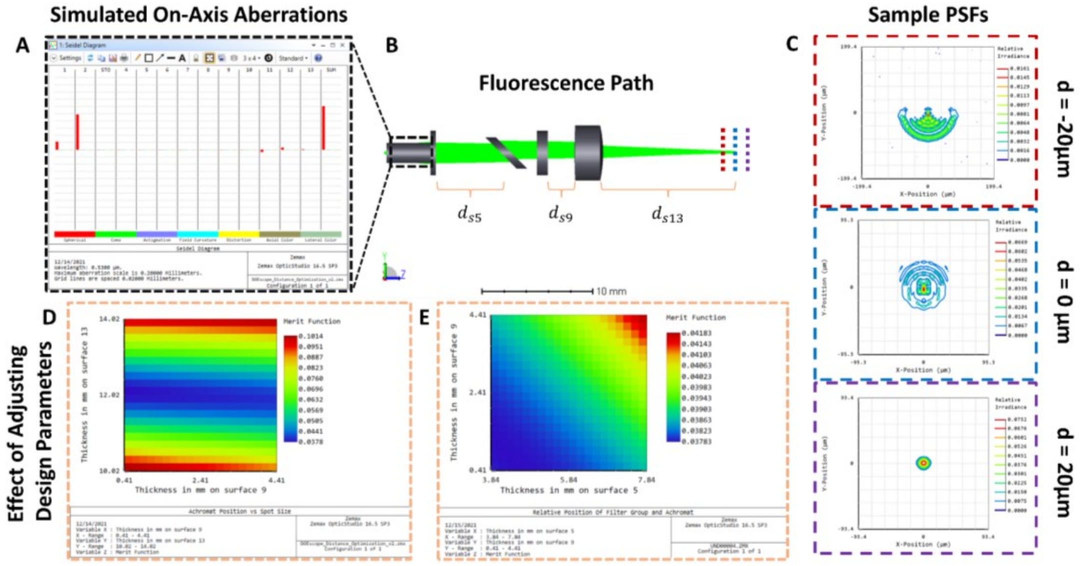

Fig. S1.2. Zemax model of a EDoF-Miniscope with Proxy Phase Mask. (**A**) We characterize the on-axis aberrations by modeling the EDoF-Miniscope in Sequential mode using publicly available Zemax files for the corresponding off-the shelf components and using the built in Seidel aberration analysis to determine the coefficients for an on-axis point source. We model our DOE as a thin piece of glass on the back surface of the GRIN lens. . (**B**) The shaded model of the emission/fluorescence path. The emitted optical signal enters the GRIN lens, passes through the glass substrate for the DOE, dichroic mirror and emission filter before being focused onto the imaging plane by a achromatic lens. The simulation considers different distances between the optical components to explore their effect of the resulting spot size as a way of exploring the design space for our miniscope. (**C**) Samples of the nominal PSF predicted by Zemax at separated axial planes. (**D**) 2D universal plot detailing the effect of changing the distance between the emission filter and achromat (surface 9) vs the achromat on the imaging plane (surface 13) on our merit function (rms spot size). (**E**) 2D universal plot detailing the effect of changing the distance between the GRIN lens and dichroic mirror (surface 5) vs the emission filter and achromat (surface 9) on our merit function (rms spot size).

Our Zemax model predicts the optical performance as well as anticipated aberrations of the EDoF-Miniscope in the sequential mode (see **Fig.S1.2A-B**). We exclusively examine the effects of the emission channel on the resulting PSF and assume that we have an ideal illumination channel (see **Fig.S1.2C**). We decouple these components so that we may explore the effects of the EDoF-Miniscope's design on the resulting PSF. The illumination channel will be dominated by the performance LED light source as collimated by an off-the-shelf drum lens. We first set the GRIN, achromatic lens and camera plane based on the effective focal lengths of each component as provided by the manufacturer. We next place an on-axis monochromatic source on the front focal plane of the GRIN lens with a central wavelength at the center of the GCamP6 spectrum (~509nm) to optimize the distances between the emission channel optics (see **Fig.S1.2D-E**). We anticipate that any chromatic aberration will be negligible since our sources are bandpass filtered and directly interrogate the effects of chromatic aberration on the resulting EDoF in simulation in **Supp. 4.3**. We utilize a 2D universal plot to compare the effect of adjusting multiple components on the RMS spot size of our PSF in a compact plot. Adjusting the position of the emission filter had a negligible effect on the RMS PSF and adjusting the achromat could be compensated for by moving the imaging plane. As a result, future iterations of the EDoF-Miniscope may be shrunk without

inhibiting the anticipated optical performance of the platform to maximize its compactness for head-mounted deployment. Importantly, Zemax predicts that, due to the strong spherical aberration, the PSF focuses before the nominal imaging plane. This reinforces our selection of a 0.23 mm working distance GRIN lens as opposed to a 0 mm working distance GRIN lens for fixed samples since the latter would truncate the focus on the platform. In our analysis, we replace the phase mask with a 0.5 mm thick glass cover slip to analyze the refractive effects of our DOE's substrate.

2 Genetic Algorithm Convergence

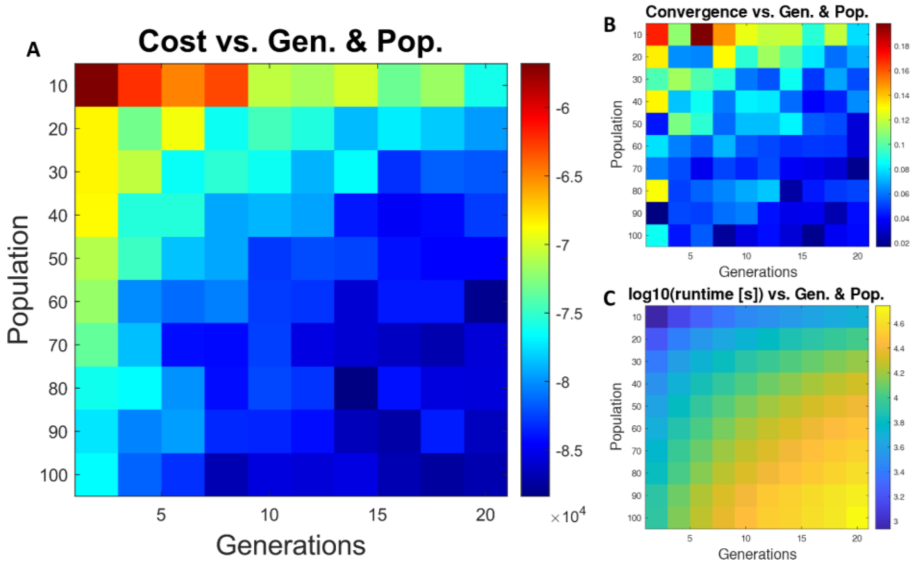

Fig. S2.1. Convergence Analysis of the Genetic Algorithm. (**A**). We analyze the fitness value (cost) produced by a binary DOE optimized by our genetic algorithm under different population sizes and generations. We run the genetic algorithm multiple under a given set of conditions to better characterize the anticipated optimized cost as well as investigate the fluctuation in the optimized cost between successive trials (see 1B). For robustness, we ran each trial 10 times and reported the mean cost. (**B**) Here, we analyzed the coefficient of variation over all trials for a given set of conditions. In general, the coefficient of variation decreases as population and generations increase, indicating that the genetic algorithm achieves more stable solutions by increasing these parameters. This allows us to anticipate that the genetic algorithm converges to more well-posed solutions for reliability as we tune these parameters. (**C**) As the population and number of generations increase, the optimization requires more total runtime on a CPU (Intel(R) Core (TM) i7-7700HQ CPU @ 2.80GHz) to complete. Here, we report the average time it takes the algorithm to complete under a set of conditions when averaged over 10 trials. The coefficient of variation for this value was negligible, leading us to use the average runtime as the sole consideration for optimization time.

Genetic algorithms navigate complex landscapes, such as designing 3D sculpted light distributions[47], by using the principles of evolution to iteratively refine the desired parameters within a population of candidate solutions using a user-defined objective function. Often modeled as Markov Chains, prior work has shown that genetic algorithms are ergodic under weak conditions and will not remain in a suboptimal minimum indefinitely regardless of the objective

function or initial conditions. However, genetic algorithms often undergo punctuated equilibrium, which can make it difficult to quantize convergence[32]. We characterize the convergence properties of our genetic algorithm by sweeping through the population size and number of generations and analyzing the mean fitness value of the optimized map over 10 iterations (see **Fig. S2.1A**). We analyze the coefficient of variation and average optimization time to better interrogate the trade space between convergence and computational cost (see **Fig. S2.1B-C**). We reasonably converge near a stable optimum in a small number of iterations (~10) with a moderate sized population of 60 candidate DOEs. While this optimization does inflate computation time, our genetic algorithm is still able to optimize a DOE in 80 min. This analysis reinforces our choice in using a genetic algorithm as a fast-paced solution for optimizing an EDoF-Miniscope for deployment in scattering media.

3 Experimental Test Setups

3.1 High Precision DOE Alignment Setup

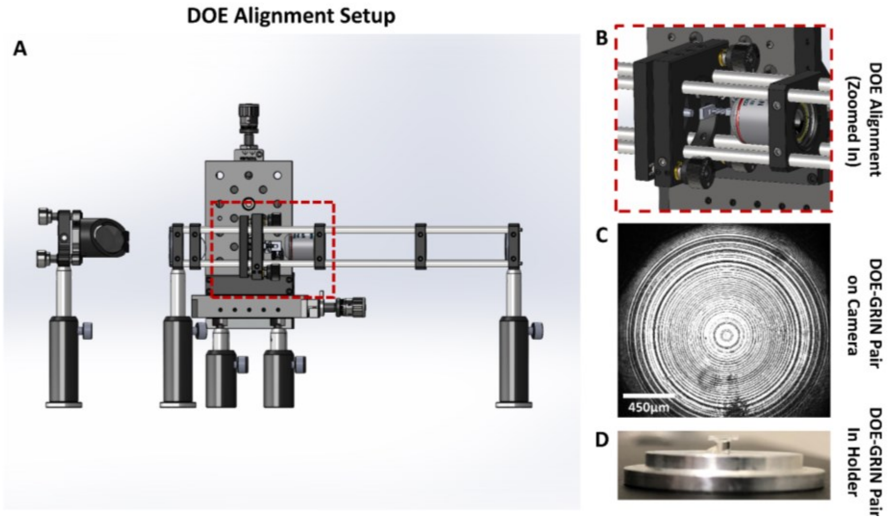

Fig. S3.1. Precision Alignment of the DOE and GRIN Lens. (**A**) Optical setup used to align the DOE and GRIN lens. The cage mounted setup aligns a collimated laser illumination source to the GRIN lens. The free space stage brings the DOE onto the optical axis and into contact with the GRIN lens to glue them together. (**B**) Zoom in on the binary DOE holder and GRIN lens to showcase the alignment procedure. (**C**) A final aligned DOE-GRIN pair as viewed from the visual feedback camera. All alignment is done under visual guidance by adjusting the DOE relative to the fixed GRIN lens. (**D**) A final aligned DOE-GRIN pair after removing it from the alignment setup.

We align the DOE to the GRIN lens using the setup shown in **Fig. S3.1A-B**. First, we collimate a laser through a spatial filter (Newport, 910A) with a 10× objective (Newport, M-10X) and a 25 μm pinhole (Newport, 910PH-25). The laser is then deflected by an elliptical mirror (Thorlabs, BBE1-E02) in a 45-degree mount (Thorlabs, H45E1) into the cage mounted setup. The cage mount contains two 4f optics systems: the miniaturized optics system and a relay optics system. The miniaturized optics system consists of a primary lens (Thorlabs, f= 25 mm, LA1951), the GRIN lens in a custom milled mount held by a 1-inch optics holder (Thorlabs, CP33) (see **Fig. S3.1D**)

and the DOE. The primary lens and GRIN lens act as a 4f system to demagnify the laser and illuminate the DOE, which is integrated into the setup through a custom holder (See **Fig. S3.1B**) and a 3-axis linear free space stage (Thorlabs, PT3A) to bring the DOE on-axis with high spatial precision. The relay system consists of an objective (Nikon, MRL00042) and tube lens (Thorlab, f = 150 mm, LA1433) and magnifies the resulting image onto a camera (FLIR, BFLY-PGE-50A2M-CS) (see **Fig. S3.1C**) to enable visual feedback for high precision alignment.

To adhere the DOE to the GRIN lens, we use the following procedure:

1. Remove all components to the left of the objective lens as shown in Fig. 3A except for the elliptical mirror and mount.
2. Remove the GRIN holder from the CP33 O1" optics mount.
3. Place a mask blank in the back slot of the GRIN holder.
4. Place the GRIN in the through hole and adhere it in place using a small droplet of dental paste.
5. Determine the top surface of the DOE (i.e. the face with the features etched in it). We use KLA Tencor Alpha Step 500 Surface Profiler, which has an oblique illumination channel and allows us to visually determine the top from bottom surface of a finished DOE.
6. Place the DOE in the DOE holder so that the surface with the pattern faces away from the GRIN lens.
7. Attach the DOE holder onto the PT3A so that it is roughly on-axis and in front of the objective lens.
8. Turn on the laser illumination and use the PT3A to bring the mask in-focus in front of the objective using visual guidance.
9. Scan the DOE along z, away from the objective lens, to ensure all components are well aligned on the optical axis and there is no noticeable drift in the DOE position on the camera.
10. Place the GRIN holder back in the CP33 mount and rebuild all components to the left of the GRIN lens. Use a level to ensure the GRIN lens is on the optical axis.
11. Physically place the CP33 mount close to the DOE such that the DOE is within the aperture of the GRIN lens but not so that the components are touching.
12. Place a small droplet of NOA63 glue on the exposed surface of the GRIN lens.
13. Use the PT3A stage to bring the DOE in contact with the GRIN lens. Since the GRIN lens might now be perfectly straight, use the x- and y-axes to keep the DOE in the center of the GRIN's aperture.
14. After achieving contact, make any last adjustments and cure the glue by illuminating the DOE-GRIN pair through the objective with the UV source.
15. Remove the CP33 Mount with the now adhered DOE - GRIN pair and integrate into an EDoF-Miniscope.

3.2 Automated Test Platform for Fixed Samples

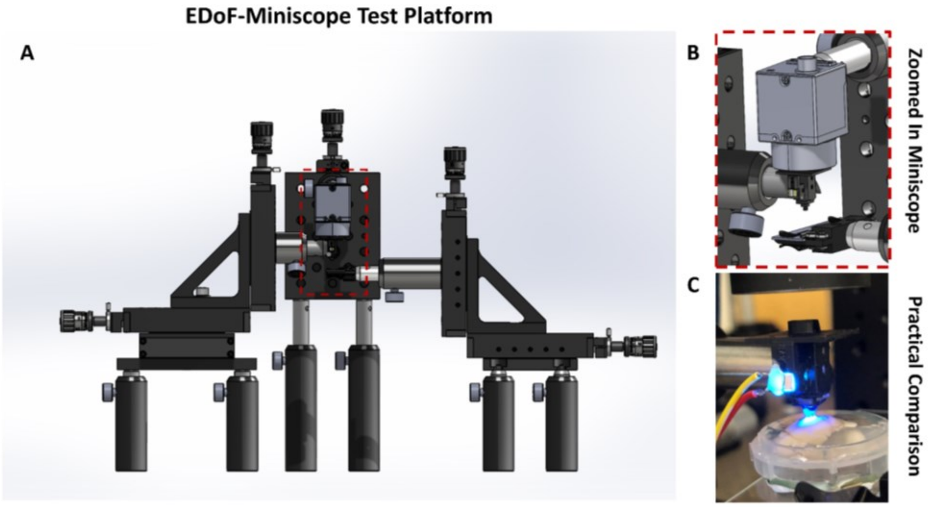

Fig. S3.2. Automated Test Platform for Interrogating Static Samples. (**A**). The test setup consists of two three-axis Thorlabs linear 1” stages (PT3A, Thorlabs) to hold the miniscope and sample respectively as well as one one-axis linear 1” stage (PT1A, Thorlabs) for the camera. The z-stage of the sample stage has been automated through Pycro-Manager to synchronize stage movement with the camera acquisition. (**B**) To achieve a high precision alignment, the EDoF-Miniscope is mounted on an O1/2” Thorlabs post which is integrated onto a PT3A stage and is manually aligned to the sample using the camera as a reference. (**C**) Example of the alignment of the EDoF-Miniscope to the whole fixed mouse brain as a practical example.

We integrated the EDoF-Miniscope into the setup described in **Fig. S3.2A** to perform our experiments on fixed samples. The setup consists of two linear three-axis stages (Thorlabs, PT3A), and a linear one-axis stage (Thorlabs, PT1A). One three axis stage adjusted the xyz position of the EDoF-Miniscope through a Thorlabs optical post fixed into the side fin of the platform. The other three-axis stage was outfitted with a sample holder (Thorlabs, MAX3SLH) to adjust the XYZ position of several fixed samples on glass slides. The one-axis stage adjusted the z position of the camera. To align the camera and miniscope, we first placed a sample of sparse 1 μm fluorescent beads under the EDoF-Miniscope and adjusted the position of the miniscope and camera until the beads were in focus, as depicted in **Fig. S3.2B.** Next, we replaced the 1 μm fluorescent beads sample with another fixed sample and used the sample stage to bring the new sample into focus. The z-axis of the sample stage was replaced with a DC actuator (Thorlabs, Z806) and automated through Pycro-Manager[34]. This allowed us to automate both the position of our sample as well as our camera acquisition to synchronize our data collection. We utilized this platform to capture single plane as well as z-scan imaging sessions of fixed samples.

4 DOE Perturbation Analysis

4.1 Effect of Lateral and Axial DOE Displacement on the DoF

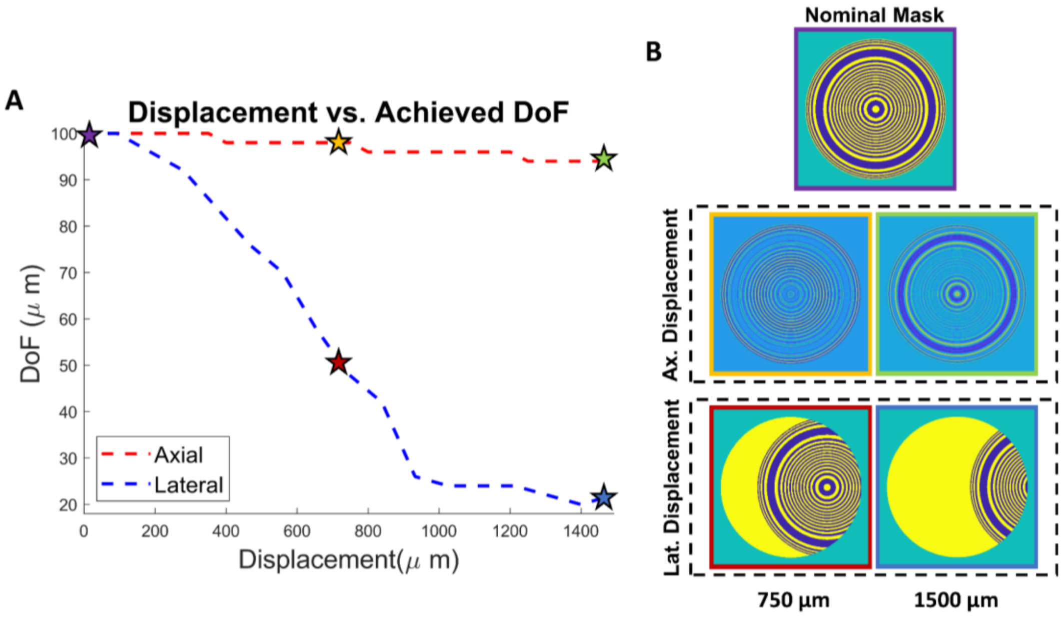

Fig. S4.1. Effect of Lateral and Axial DOE Displacement on the DoF. (**A**) Achievable DoF by our EDoF-Miniscope when the DOE is displaced axially (red line) and laterally (blue line) on the pupil plane. (**B**) Examples of the resulting pupil function without modification (top row) when the DOE is axially displaced by 750 μm and 1500 μm (middle row) and when laterally displaced by 750 μm and 1500 μm (bottom row).

We characterize the effects of axially and laterally displacing our DOE on the DoF in simulation to analyze our alignment tolerances when assembling the EDoF-Miniscope in **Fig.S4.1A**. We utilize **Eq. S1** to simulate the 3D intensity distribution, where *M(u,v)* is replaced by our displaced mask. To perform lateral displacement, we first utilize the Matlab circshift command in Matlab to shift our mask on a pixelated grid by the nearest integer number of pixels to reach a desired displacement. To perform axial displacement, we utilize the defocus kernel described in **Eq. S2** to simulate the optical field generated by an axially displaced mask when backpropagated to the pupil plane. We find that axial displacement is a weak parameter on the resulting DoF. We predict that, in a miniscope, any axial displacement will be small compared to the size of the DOE. In addition, the rings that comprise the DOE vary in size between ~4 μm and ~150 μm. While small rings may blur as the DOE defocuses, **Fig.S4.1B (middle)** shows that many of the larger structures are still perceivable within our simulated range.

We find that the mask is more sensitive to lateral displacement. Intuitively, we may model small lateral shifts as convolutions of our DOE with an off-axis delta function, which will result in sheared versions of our PSF in any cross-sectional plane but should not significantly affect the achieved DoF. However, as we continue to laterally move the DOE, the DOE pattern is increasingly clipped by the aperture and replaced by the nominal bare aperture of the GRIN lens (**Fig.S4.1B (bottom)**). As this occurs, we expect the DoF to significantly decrease and ultimately produce the nominal DoF, which is 20 μm for this given simulation. From this analysis we may conclude that our DOE is negligibly affected by our reported lateral alignment accuracy (70 μm) and any deviation between the thickness of our substrate (500 μm) and the working distance of the GRIN lens (230 μm).

We simulated all displacements for fixed parameters: n = 1.33, l_s = 100 µm, for 5 µm on-axis sources, over a 120 µm range with a 2 µm step size. We characterize DoF using the number of depth planes that contain an intensity value within 50% the maximum 3D value.

4.2 Alignment Precision under Visual Guidance

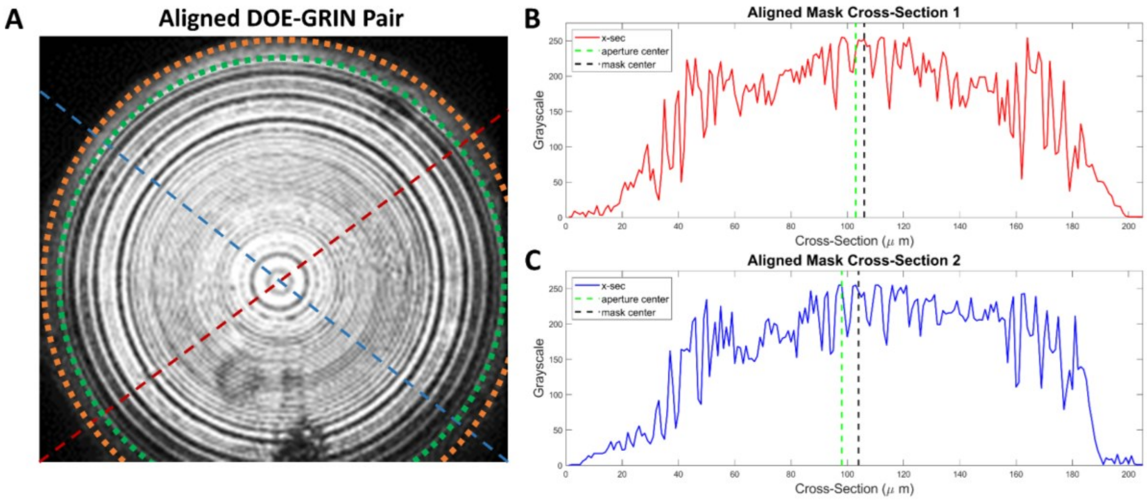

Fig. S4.2. Achieved Precision under Visual Alignment. (**A**). Example feedback image when visually aligning the DOE to the GRIN lens using the setup in **Fig. S3.1A**. The two cut lines correspond to (**B**) and (**C**) respectively. The green circle marks the edge of the DOE and the orange circle marks the cutoff of the aperture. We may use the difference in the geometric center of the DOE and GRIN aperture to characterize the alignment.

After performing visual alignment, we characterize the lateral precision of our alignment of our DOE - GRIN pair to confirm that our setup can achieve a precision within the tolerances described by **Supp. 4.2**. We characterize our alignment by comparing the geometric center of the DOE to the geometric center of the surrounding GRIN aperture (See **Fig. S4.2A**). The center of the DOE is easily identifiable by the structure of the center ring. We determine the cutoff of the aperture as the closest pixels on either side of the DOE that reach a grayscale value of 0. We can then determine the center of the aperture as the midpoint between those coordinates and compare that calculated value to the midpoint of the DOE to determine our alignment accuracy. For robustness, we perform that analysis over multiple cutlines (See **Fig. S4.2B-C**) to determine that we have a *maximum* lateral misalignment of 70 µm. The material list for the high-precision alignment setup as well as CAD models for the custom components can be found on our GitHub.

4.3 Phase Shift Error versus Deployed EDoF

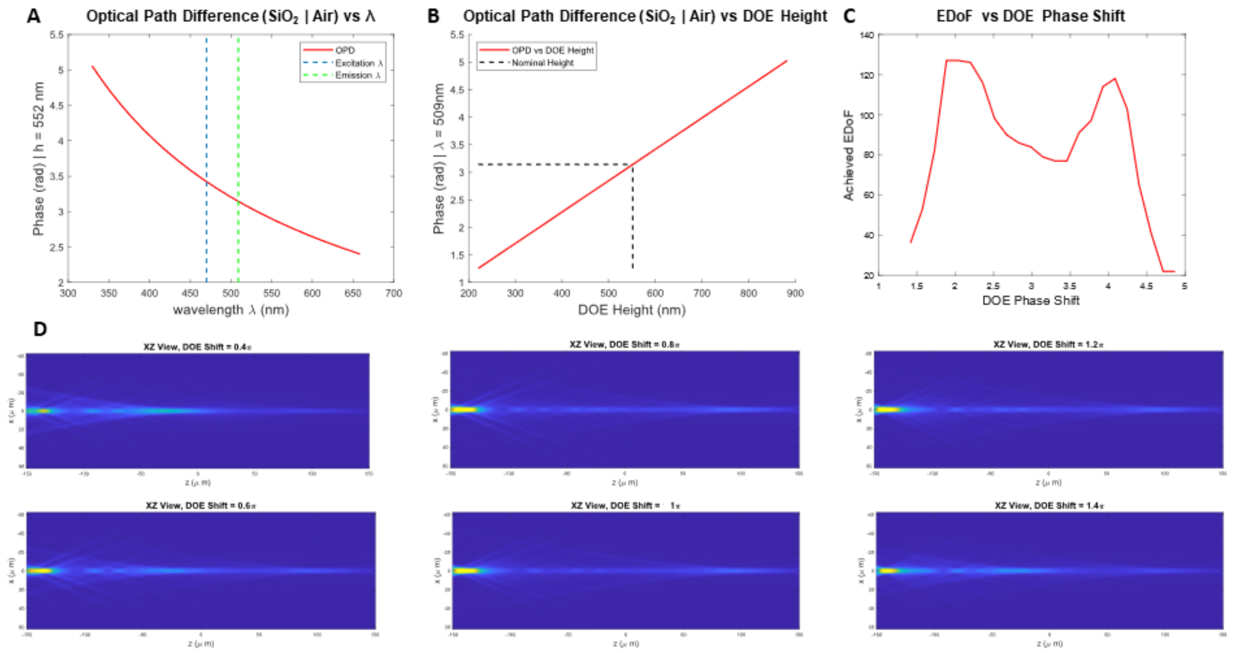

Fig. S4.3. Phase Shift Error on Induced DoF. (**A**). Phase shift induced by our DOE etched on fused silica when illuminated by different wavelengths. Our DOE is optimized to produce a π phase shift for the emission wavelength and deviates by ~11% when illuminated by the excitation wavelength. (**B**) Phase shift induced by our DOE under different etched heights (λ = 509nm). (**C**) Simulated depth of field, as determined by the number of depth planes which contain 50% or more of the peak intensity within the 3D volume, under different DOE phase shifts. (**D**) Cross-section of the simulated 3D point spread function under different phase shifts induced by our DOE.

To characterize the effects of wavelength deviation and etch error on our DOE, we interrogate the resulting 3D PSF from our binary DOE when it induces a non-ideal phase shift in simulation. First, we determine the wavelength dependence of the refractive index for fused silica[48]. Using this relationship, we simulate the optical path difference induced by our DOE for a fixed height, optimized for the central emission wavelength (h = 552nm, λ = 509nm) over the visible range (**Fig. S4.3A**). Next, we repeat the same simulation for a fixed wavelength (λ = 509nm) but variable etch heights to simulate the effects of etch error on the induced optical path difference (**Fig. S4.3B**). Using the forward model described in **Eq. S1**, where *M(u,v)* is replaced with a DOE that induces a non-ideal phase shift, we simulate the resulting EDoF (**Fig.S4.3C**). Here, the EDoF is described as the total number of depth planes within our simulated 3D volume that contains a maximum intensity value within 50% of the peak value. We simulate our 3D PSF for varying DOE phase shifts (**Fig. S4.3D**) and find that as our phase shift deviates from π, we redistribute the power coupled into specific orders of diffraction. Previous work has characterized how much power will be contained in a specific diffraction order as a function of DOE phase shift[49]. While generating more orders of diffraction has the effect of concentrating optical power at more locations along the optical axis, this leads to two detriments. First, less power will be contained within each individual order of diffraction meaning that more power will be concentrated in the defocused portion of the total optical field at each axial location. Second, as the phase shift approaches 0 and 2π, our DOE will contrate more optical power within the zeroth unmodulated order of diffraction thus limiting

the EDoF closer to the nominal DoF of the miniscope. As a result, ensuring that our DOE phase shift as close to π as possible will ensure that our DOE produces high contrast and extended foci.

5 EDoF-Miniscope Characterization on Fluorescent Samples

5.1 Axial Elongation Within a 5 μm Bead Scattering Phantom

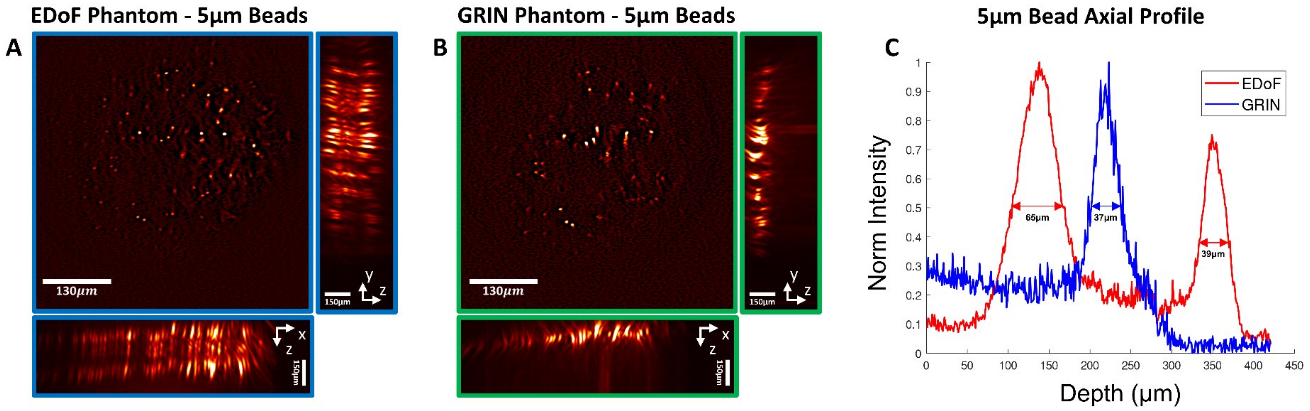

Fig. S5.1. Axial Elongation Within a 5 μm Bead Scattering Phantom. (**A**) Volumetric information from an axial scan of a scattering phantom (l_s = 100 μm) embedded with 5 μm beads imaged by the EDoF-Miniscope. The center image is one in-focus plane from the +1st diffraction order and the side and bottom images are xz and yz MIPs respectively. (**B**) Volumetric information from an axial scan of the same scattering phantom imaged by a miniscope. The center image is one in-focus plane from the +1st diffraction order and the side and bottom images are xz and yz MIPs respectively. (**C**) Axial profile from cut line on MIP for the EDoF-Miniscope and miniscope.

We characterize the axial elongation of 5 μm fluorescent beads in a scattering phantom (l_s = 100 μm) as a proxy for neurons in neural tissue. After using the setup described in **Supp. 3.2** to bring the sample into focus for the EDoF-Miniscope and miniscope, we utilize an automated sample stage to acquire a z-stack. We show the one focal plane image combined with xz and yz MIPs for the EDoF-Miniscope (see **Fig. S5.1A**) and the miniscope (see **Fig. S5.1B**) respectively. We plot the cut lines from **Fig. S5.1A-B** in **Fig. S5.1C** to characterize the axial elongation. The EDoF-Miniscope achieves an axial elongation of 107 μm between the two foci while the miniscope achieves an axial elongation of 37 μm. We notice that the recovered EDoF for both the EDoF-Miniscope and miniscope exhibit aberration, in particular at the peripheries of the FoV. We hypothesize that these aberrations arise from off-axis aberrations of the GRIN lens not considered during optimization as well as alignment imperfections in the GRIN lens due to manual assembly.

5.2 Axial Elongation Within a 10 μm beads Scattering Phantom

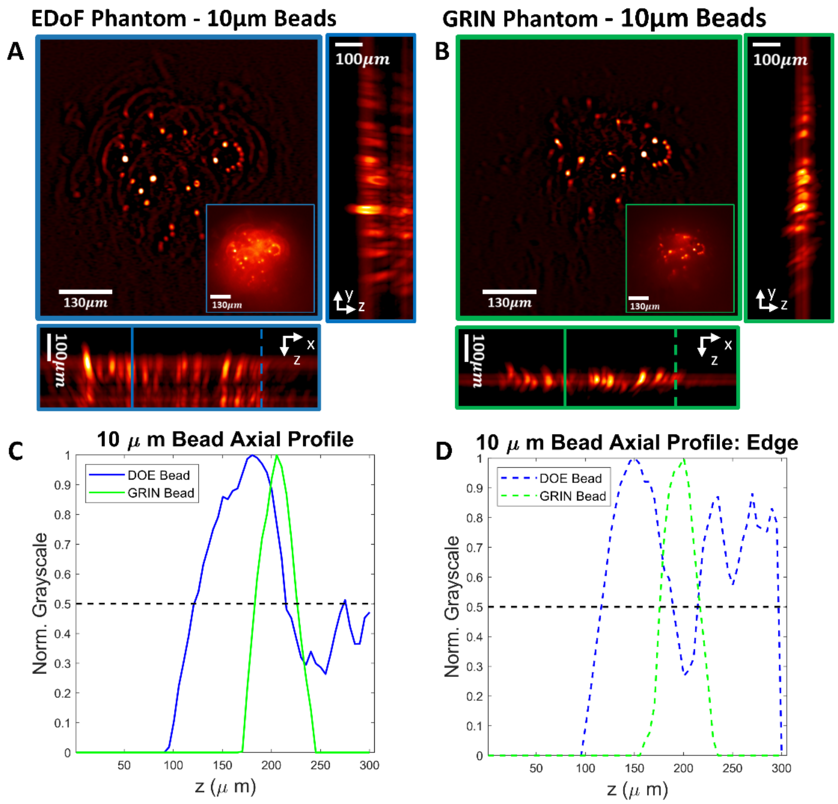

Fig. S5.2. Axial Elongation Within a 10 µm Bead Scattering Phantom. (**A**) Volumetric information from an axial scan of a scattering phantom (l_s = 100 µm) with 10 µm beads imaged by the EDoF-Miniscope. The center image is one in-focus plane from the +1st diffraction order and the side and bottom images are xz and yz MIPs respectively. (**B**) Volumetric information from an axial scan of the same scattering phantom imaged by a miniscope. The center image is one in-focus plane from the +1st diffraction order and the side and bottom images are xz and yz MIPs respectively. (**C**) Cut lines near the center of the FoV from the EDoF-Miniscope and miniscope. (**D**) Cut lines near the edge of the FoV from the EDoF-Miniscope and miniscope.

We characterize the axial elongation of 10 µm fluorescent beads in a scattering phantom (l_s = 100 µm) as a proxy for large neurons in neural tissue. After using the setup described in **Supp. 3.2** to bring the sample into focus for the EDoF-Miniscope and miniscope, we utilize an automated sample stage to acquire a z-stack. We show the one focal plane image combined with xz and yz MIPs for the EDoF-Miniscope (see **Fig. S5.1A**) and the miniscope (see **Fig. S5.1B**) respectively. We plot the cut lines from **Fig. S5.1A-B** in **Fig. S5.1C** to characterize the axial elongation. The EDoF-Miniscope achieves an axial elongation of 95 µm in the first order near the center of the FoV and 85 µm near the edge of the field of view. The miniscope achieves an axial elongation of 45 µm in the first order near the center of the FoV and 40 µm near the edge of the field of view.

5.3 SBR Analysis Over 5 µm Bead Phantoms of Varying Density

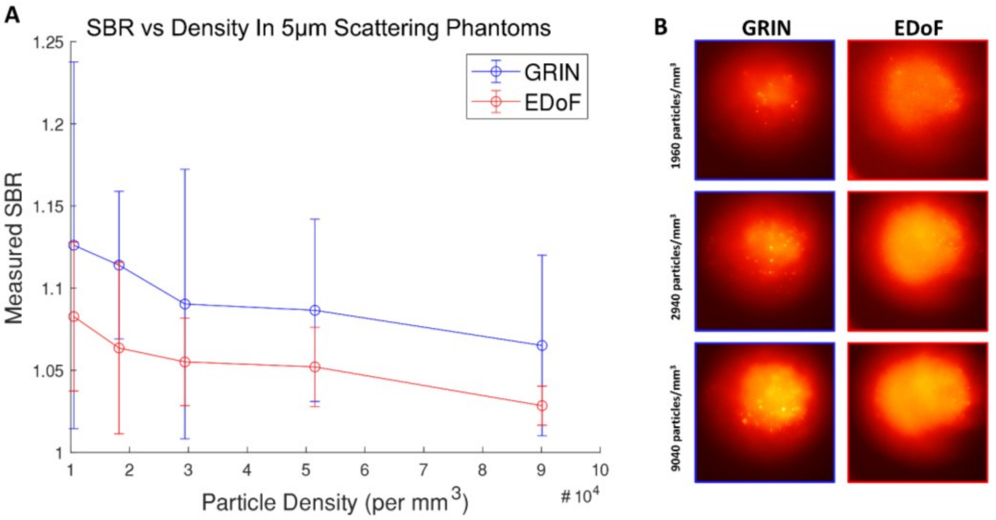

Fig. S5.3. SBR Analysis Over 5 µm Bead Phantoms of Varying Density. (**A**) Signal-to-Background ratio averaged over 10 particles across the FoV of several phantoms of varying density as imaged by the EDoF-Miniscope and a miniscope. (**B**) Example images from the miniscope (left) and EDoF-Miniscope (right) of several phantoms of different densities.

To investigate any reduced contrast in the EDoF-Miniscope, we imaged 5 µm bead scattering phantoms of controlled densities (~1000-10000 particles/mm^3) with both the EDoF-Miniscope and a miniscope. We randomly determined the centroids of 10 particles across the FoV of each image and determined the signal by averaging an 8 pixels circular region-of-interest around each centroid and determined the background by averaging a 35 pixels circular region-of-interest. The mean SBR and standard deviation were determined for each density (see **Fig. S5.3A**). In general, the EDoF-Miniscope exhibits a 4% decrease in SBR over all phantoms (see **Fig. S5.3B**).

6 Analysis of Post-Processing Filter

6.1 Simulated Post-Processing Performance

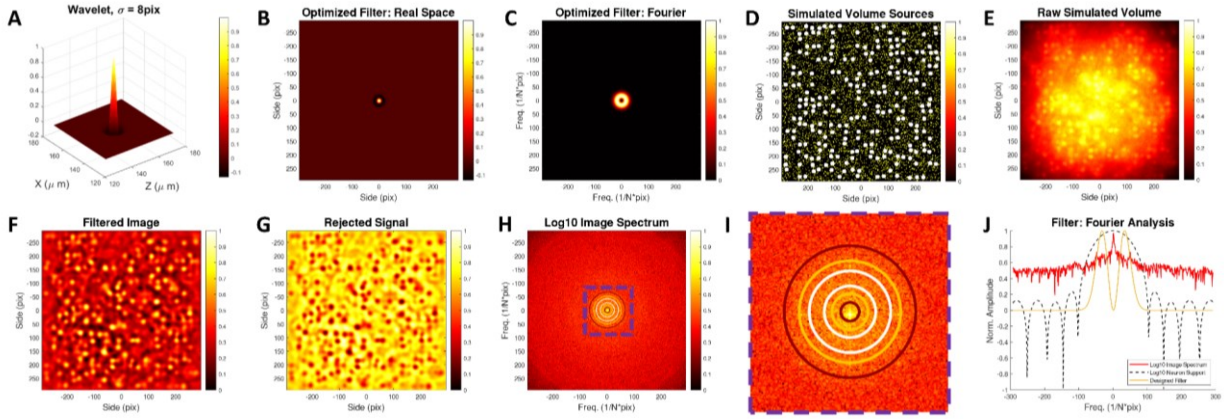

Fig. S6.1. Simulated Post-Processing Filter. (**A**) A surface profile of the filter utilized in post-processing static neuronal frames. (**B**) Real space convolution filter and (**C**) its profile in the fourier domain. (**D**) The MIP of a simulated test volume consisting of 5 µm neurons and 1 µm background particles. (**E**) The simulated raw

measurement when the simulated volume was distributed over 150 μm. (**F**) The post-process framed and (**G**) the rejected information from applying the post-processing filter. (**H**) An overlay of the filter Fourier spectrum (contour) on the spectrum of the simulated image (image) with (**I**) a zoom-in on the spectrum and (**J**) a cross-section of the image spectrum, Fourier space filter and the spectrum of a 5 μm proxy particle.

We analyze the performance of the filter used for post-processing our fixed samples, following a proposed form[27] (see **Fig. S6.1A**). We found that utilizing a Laplacian of Guassian (LoG) filter with a standard deviation of 8 pixels performs adequate background suppression while extracting neuronal structures (see **Fig. S6.1B**). Intuitively, the LoG filter is an annulus in Fourier space, reinforcing its ability to suppress constant and slowly varying background as well as select objects within a certain band (see **Fig. S6.1C**). We simulated a neuronal volume while imaging with an EDoF-Miniscope with 5 μm proxy neurons with 1 μm sources to artificially add background (see **Fig. S6.1D-E**). Applying the filter extracts the 5 μm proxy particles while suppressing the background and 1 μm sources (see **Fig. S6.1F-G**). We overlay the Fourier transform of the designed filter on the spectrum of the simulated image to emphasize that the post-processing filter is tailored to extract the target particles (see **Fig. S6.1H-J**).

6.2 Investigating Filter Performance on Simulated 5 μm Sources

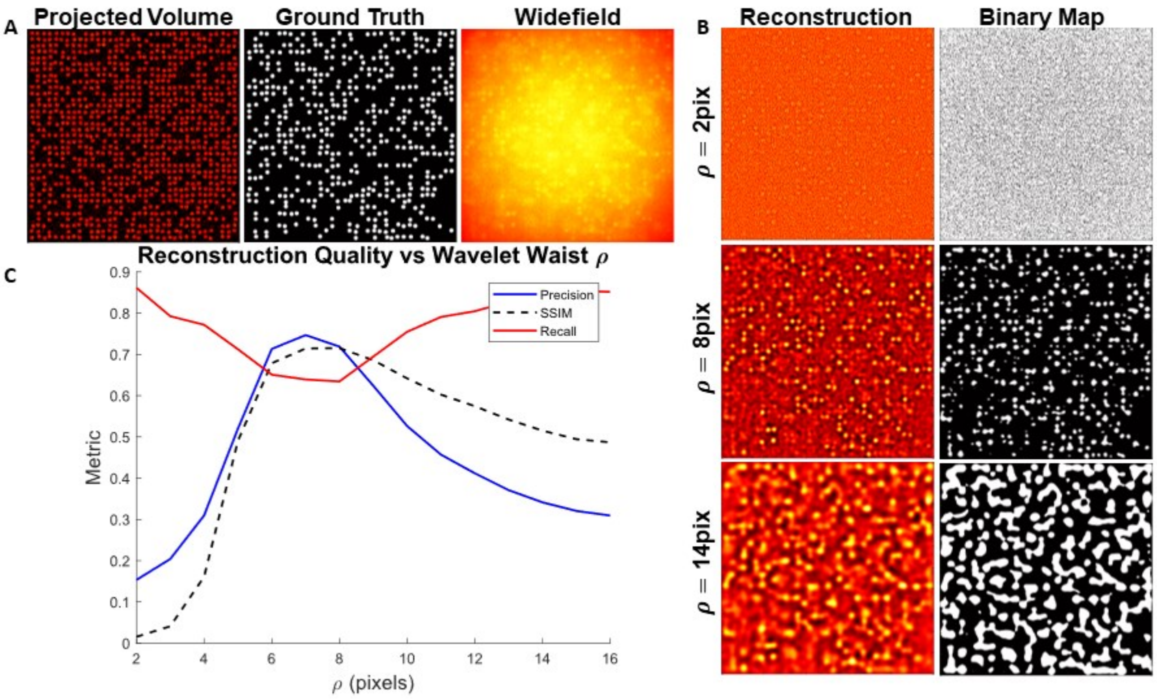

Fig. S6.2. Investigating Filter Performance on Simulated 5 μm Sources. (**A**) Projection of a simulated population of 5 μm neurons distributed within a 300 x 300 x 300 μm^3 volume (left). We truncate the projection to a 100 μm depth range to separate our ground truth neurons that we expect to recover with the EDoF-Miniscope and background neurons outside the induced EDoF (middle). Widefield image generated by the forward model described in **Eq. S3** using our optimized binary DOE (right). (**B**) Filtered images produced by applying the LoG filter to our widefield image with different underlying gaussian waists (left) as well as their binarized counterparts used to determine the accuracy of the reconstruction (right). (**C**) Analysis of the recovered frames after post processing. We

compare three metrics: precision, recall and SSIM to quantize the effectiveness of the LoG filter in extracting the simulated neurons.

We quantize the effectiveness of the LoG filter in extracting neurons from a low SBR and noisy scene in simulation by first generating a 300 x 300 x 300 μm^3 volume of 5 μm neuronal-like sources. Our simulated grids and environments match the simulation used to optimize the EDoF-Miniscope. Since our induced EDoF is optimized to cover 100 μm for 5 μm sources we use this value as our cutoff between "in-focus" and "background" neurons in our analysis. We show the projected volume of neuronal sources, the recoverable sources (a binary ground truth) and corresponding widefield simulation using the whole volume in **Fig. S6.2A**. Next, we apply successively broader LoG filters to the widefield image and quantize the recovery of the in-focus sources by binarizing the frames (min-max normalized, threshold = 0.3) and calculating the precision, recall and SSIM between the binarized frame and the ground truth. We show example reconstructions and their corresponding binarizing in **Fig. S6.2B**. We present the finalized metrics as a function of LoG gaussian waist in **Fig. 6.2C**. We discover that we achieve the best recovery and quality in a waist range between 5-9 pixels.

6.3 Investigating Filter Performance on Simulated 10 μm Sources

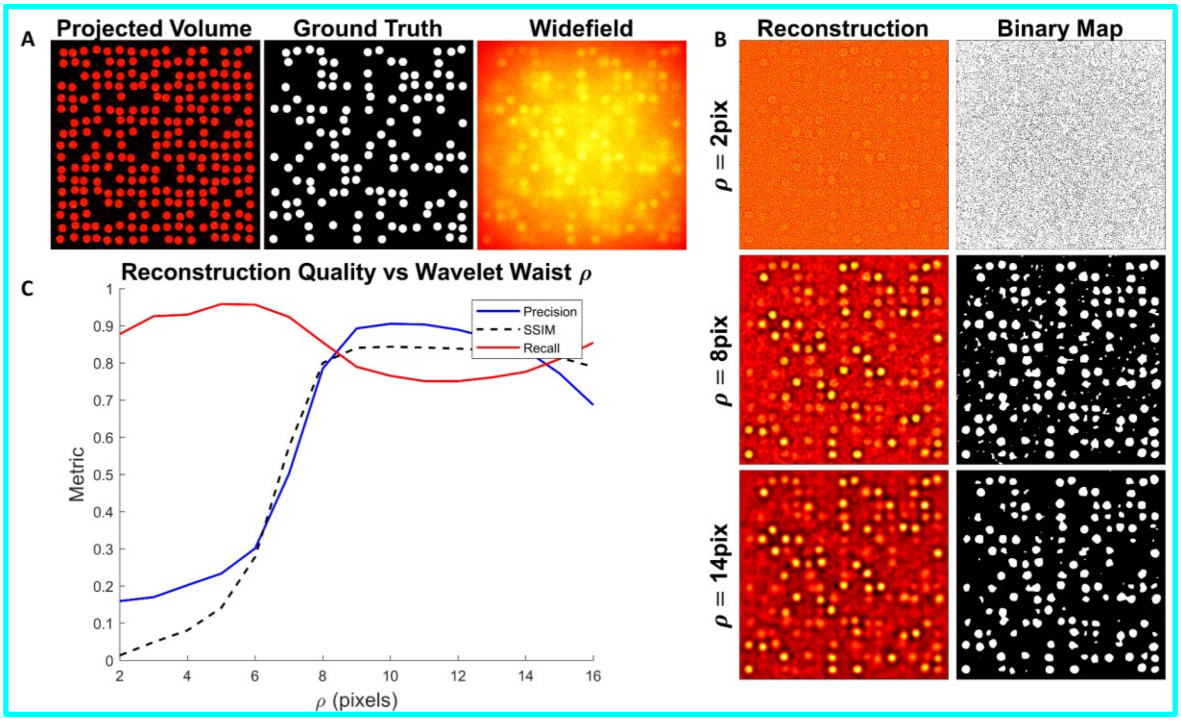

Fig. S6.3. Investigating Filter Performance on Simulated 10 *μm* Sources. (**A**) Projection of a simulated population of 10 μm neurons distributed within a 300 x 300 x 300 μm^3 volume (left). We truncate the projection to a 100 μm depth range to separate our ground truth neurons that we expect to recover with the EDoF-Miniscope and background neurons outside the induced EDoF (middle). Widefield image generated by the forward model described in **Eq. S3** using our optimized binary DOE (right). (**B**) Filtered images produced by applying the LoG filter to our widefield image with different underlying gaussian waists (left) as well as their binarized counterparts used to determine the accuracy of the reconstruction (right). (**C**) Analysis of the recovered frames after post processing. We

compare three metrics: precision, recall and SSIM to quantize the effectiveness of the LoG filter in extracting the simulated neurons.

We quantize the effectiveness of the LoG filter in extracting neurons from a low SBR and noisy scene in simulation by first generating a 300 x 300 x 300 μm^3 volume of 10 μm neuronal-like sources. Our simulated grids and environments match the simulation used to optimize the EDoF-Miniscope. We use 100 μm as our cutoff between "in-focus" and "background" neurons in our analysis. We show the projected volume of neuronal sources, the recoverable sources (a binary ground truth) and corresponding widefield simulation using the whole volume in **Fig. S6.3A**. Next, we apply successively broader LoG filters to the widefield image and quantize the recovery of the in-focus sources by binarizing the frames (min-max normalized, threshold = 0.3) and calculating the precision, recall and SSIM between the binarized frame and the ground truth. We show example reconstructions and their corresponding binarizing in **Fig. S6.3B**. We present the finalized metrics as a function of LoG gaussian waist in **Fig. 6.3C**. We discover that we achieve the best recovery and quality in a waist range between 8-16 pixels.

6.4 Comparison to Deconvolution - Brain Slice Sample

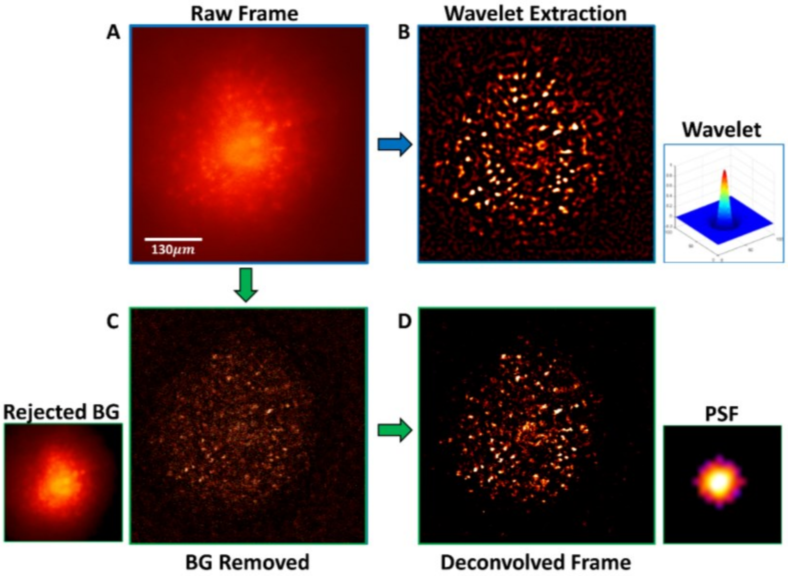

Fig. S6.4. Comparison of Filtering to Deconvolution on Neural Slices. (**A**) Raw image acquired by imaging a 100 μm fixed mouse brain slice with an EDoF-Miniscope. (**B**) EDoF-Miniscope image after the post-processing filter. (**C**) EDoF-Miniscope image after morphological background removal and (**D**) deconvolution.

We show that filtering visually outperforms standard deconvolution on a 100 μm fixed mouse brain slice. We first capture a raw frame of a 100 μm fixed mouse brain slice by the EDoF-Miniscope (see **Fig. S6.4A**). We process the raw frame with a LoG filter with a waist of 8 pixels (see **Fig. S6.4B**). We compared the filtered image to an image that was post-processed with morphological background removal[14] and deconvolution with the PSF from **Fig.1D** and Tikhonov regularization (see **Fig. S6.4C-D**). Filtering performs more effective extraction of the fixed neurons across a larger FoV when compared to the deconvolved image. We predict that the filter

is effective in two parts. First, the filter is summable to zero, allowing it to reject high background more effectively. Second, the filter exhibits a gaussian peak which averages several pixels and reduces noise. Altogether, these properties allow the filter to effectively extract neuronal signals from low contrast, high noise images.

6.5 Comparison to Deconvolution - Vasculature

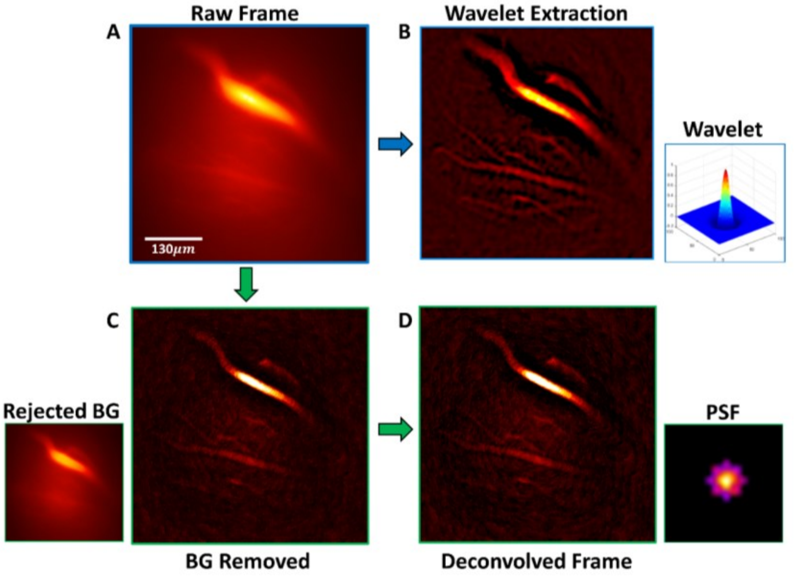

Fig. S6.5. Comparison of Filtering to Deconvolution on Stained Vasculature. (**A**) Raw image acquired by imaging stained vasculature in a whole mouse brain with an EDoF-Miniscope. (**B**) EDoF-Miniscope image after the post-processing. (**C**) EDoF-Miniscope image after morphological background removal and (**D**) deconvolution.

We show that filtering visually outperforms standard deconvolution on stained vasculature in a whole fixed mouse brain. We first capture a raw frame with the EDoF-Miniscope (see **Fig. S6.5A**). We process the raw frame with a LoG filter with a standard deviation of 8 pixels (see **Fig. S6.5B**). We compared the filtered image to an image that was post-processed with morphological background removal and deconvolution with the PSF from **Fig.1D** and Tikhonov regularization (see **Fig. S6.5C-D**). Filtering performs more effective extraction of the vasculature across a larger FoV when compared to the deconvolved image. Notably, the morphological background removal and deconvolution fail to recover the small vasculature present in the bottom of the image and artificially constrain the width of the large vessels at the top of the image. This trial emphasizes that the post-processing filter is an effective tool at extracting a variety of neural signals and generalizes across several source geometries.

7 Exploration of DOE Optimization Under Different Conditions

7.1 DOE Optimization for Single Focus Extension

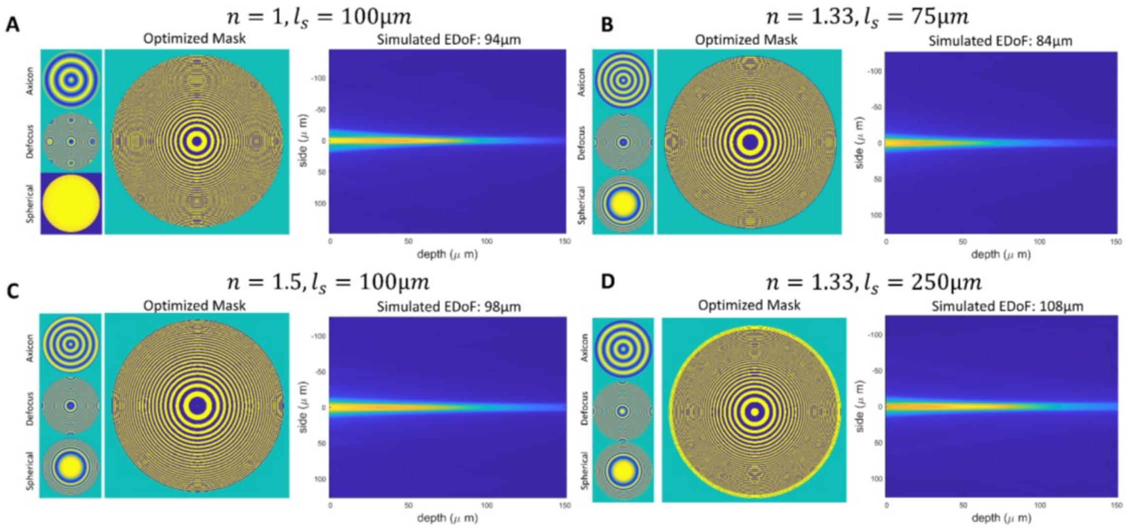

Fig. S7.1. DOE Optimization for Single Focus Extension. The learned optimized phase basis (left), binarized mask (center) and the +1st diffraction order elongation (right) for a media with **(A)** an index near air (n = 1) and similar scattering to our original case (l_s = 100 µm). (**B**) an index near water (n = 1.33) and stronger scattering to our original case (l_s = 75 µm). (**C**) an index near oil (n = 1.5) and similar scattering to our original case (l_s = 100 µm). (**D**) an index near water (n = 1.33) and similar scattering to our original case (l_s = 250 µm).

One major challenge of deploying EDoF-Miniscope in-vivo is adjusting our design to accommodate zero working distance lenses, which subsequently suppresses the -1st order of diffraction. Here, we present the generalizability of our proposed framework to optimize a single focus for a zero working distance lens in scattering media to showcase the broad appeal of using diffractive optics. For simplicity, we optimize these results over the aberrations we characterized in **Supp. 1.2** but assume that the GRIN lens now has zero working distance. Future work will explore the unique aberrations generated by zero working distance GRIN lenses.

All cases choose to elongate a 5 µm on-axis source to a target EDoF of 100 µm with the same basis and bounds as the DOE presented in the main text. We first analyze the effect of changing the assumed index of refraction while retaining a comparable scattering length to our original design (See. **Fig. S7.1A, C**). In both cases, we can achieve an axial elongation approaching the target depth. Next, we fixed the index and changed the scattering length to shorter than (See **Fig. S7.1B**) and longer than (See **Fig. S7.1D**) the original design. As expected, the DOE designed for a longer scattering length achieves the desired axial elongation while the DOE designed for a shorter scattering length was unable to achieve an elongation significantly past one scattering length. Intuitively, this makes sense as the optical intensity decays to under 36% after one scattering length, increasing the difficulty to maintain a relative intensity within 50% of the peak value (our definition of axial elongation). As a result, we confirm that our genetic algorithm is well generalizable for different media however is fundamentally limited by the scattering length. It should be noted that the mask optimized in air unlearned spherical aberration while all other masks learned a unique combination of all three basis functions. This indicates that the optimal mask is depending on the user defined conditions and no one mask generalizes across different cases. This reinforces the need for a rapid algorithm that can optimize DOEs for EDoF imaging across a variety of applications.

7.2 Exploration of Model Mismatch

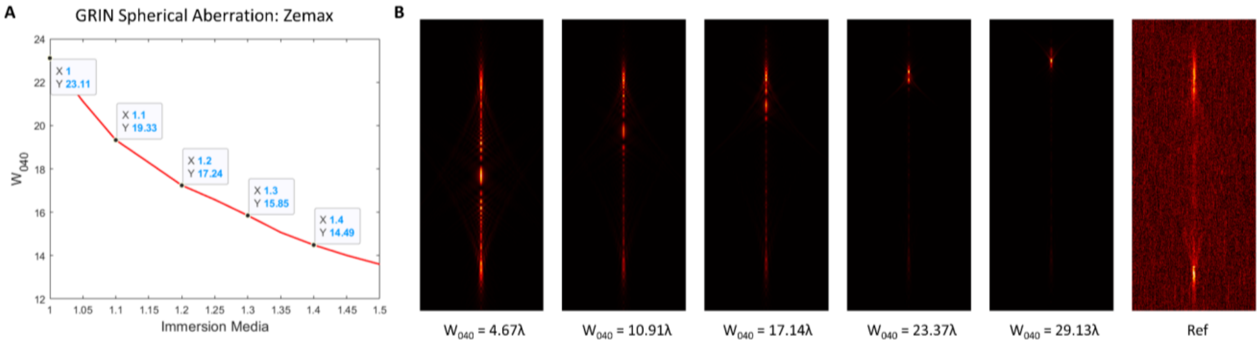

Fig. S7.2. Exploration of Model Mismatch. (**A**) We first updated and investigated our GRIN lens Zemax model to investigate how our primary aberration changes as a function of immersion media. (**B**) We adjust the assumed spherical aberration of the GRIN lens to monitor the effect on the simulated EDoF ranging from a 4.67λ (left) to 29.13λ when compared to our experimentally captured cross-section (right).

First, we analyzed the Zemax model of our GRIN lens to predict how spherical aberration changes as we insert the GRIN lens into different immersion media. In general as the index increases we observe a subsequent decrease in spherical aberration. We additionally analyze the need for more rigorous physical characterization of the GRIN lens by simulating the XZ cross-section of the EDoF-Miniscope without scattering under different assumed spherical aberrations between z = -186 µm and 160 µm to match the experimentally captured axial scan. Notably, the captured PSF exhibits foci of near equal strength which differentiates from our simulated PSFs at high spherical aberration. This indicates that our GRIN lens differs from our assumed value of 29.4λ from our Zemax simulation and is most likely closer to 10-17λ. In our future work, we will explore more comprehensive strategies to physically characterize the aberrations of the GRIN lens outside of simulation.

7.3 DOE Optimization of Twin Foci to Push Possible EDoFs

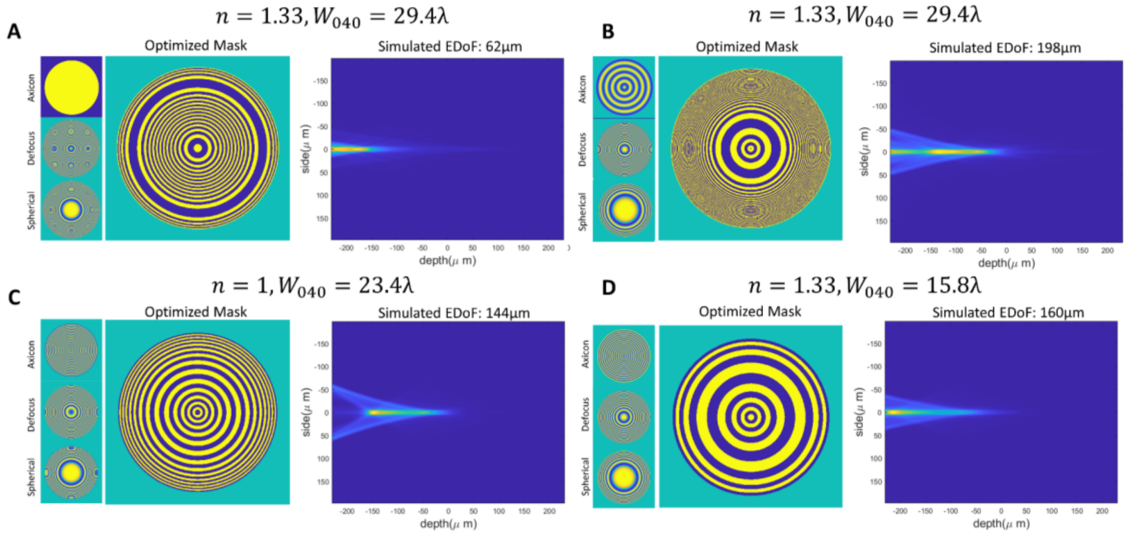

Fig. S7.3. DOE Optimization of Twin Foci to Push Possible EDoFs. Here we present an updated genetic algorithm that learns competitive EDoFs under a variety of physical assumptions. (**A**) our original mask simulated under the conditions of the brain. (**B**) A mask learned by our genetic algorithm under our originally assumed conditions. (**C**) A mask learned under the updated GRIN assumptions for air. (**D**) A mask learned under the updated GRIN assumptions for neural imaging. All simulations were conducted with a set scattering length of 100 µm.

We readapted our genetic algorithm in two key capacities: 1.) expanding the learning depth from -100 µm to 100 µm to -230 µm to 230 µm and 2.) greatly expanded the bounds for the genetic algorithm to include a broader range of phase terms to push our algorithm to learn competitive EDoF in scattering media. We compare these results to our original design (**S7.3A**). We notice that our original design minimized the importance of the -1st order by driving it outside of our simulated depth range thus encouraging our algorithm to prioritize on extending the 1st order. Here, we present several alternative DOEs that exhibit a contiguous EDoF that better merge the twin foci under these refined conditions. We first present an optimized DOE under the original GRIN lens parameters (**S7.3B**) then present two additional masks that are optimized under the updated GRIN lens parameters presented in **Supp 7.2** (**S7.3C-D**) with these expanded bounds.

These results lead to three important observations. Firstly, by allowing our algorithm to learn over an enlarged volume and bounds we are better able to account for the full optical profile of our EDoF thus allowing the algorithm to optimize binary DOEs that make more effective use of the twin foci. Interestingly, optimizing over this enlarged space tends to merge the two foci together into a contiguous EDoF close to the surface of the GRIN lens (z=-230 µm). This result tends to elongate the EDoF beyond the range of the single focus designs presented in **Supp 7.1** as well as assists in light efficiency for deployment in scattering media. Second, the resulting DOEs tend to exhibit higher phase parameters in their optimized parameters when optimized over high assumed native spherical aberration. Intuitively, the high native spherical aberration within the GRIN lens is difficult to balance between the generated twin foci, thus encouraging the mask to learn more aggressive phase parameters to balance the generated EDoF. This also has the potential effect of making these DOEs more difficult to fabricate in practice. We also noticed that third, when compared to the original design **S7.3B-D** exhibit more noticeable side lobes in the cross section.

Since our presented EDoF-Miniscope degrades SBR in exchange for its EDoF we suggest that there is a more complex tradespace between maximizing the EDoF achieved by a binary DOE and minimizing the SBR deterioration generated by side lobes and non-imaging diffraction orders in an optimized design. We intend to explore this direction in future work.

7.4 Learned DOE Parameters

Mask	Axicon (10^{-4})	Defocus (10^{-4})	Sph. Ab. (10^{-4})	Achieved EDoF (µm)	W040(λ), n, l_s(µm)
Main Text	0	1.222	0.989	58	29.4, 1, 100
S7.1A	0.054	-0.9595	-0.2163	94	29.4, 1, 100
S7.1B	0.088	-0.9628	-0.6787	84	29.4, 1.33, 75
S7.1C	0.082	-0.9965	-0.7596	98	29.4, 1.5, 100
S7.1D	0.068	-0.9145	-0.7703	108	29.4, 1.33, 250
S7.3A	0	1.222	0.989	62	29.4, 1.33, 100
S7.3B	-0.287	-0.8664	0.900	198	29.4, 1.33, 100
S7.3C	0.3283	0.5213	0.7780	144	23.4, 1, 100
S7.3D	0.2462	0.5740	0.6768	160	15.8, 15.8, 100

Table S7.4. Learned DOE Parameters. Here, we present the learned phase basis and optimization parameters used by the genetic algorithm across all trials presented in the paper.
